# Widespread parainflammation in human cancer

**DOI:** 10.1186/s13059-016-0995-z

**Published:** 2016-07-08

**Authors:** Dvir Aran, Audrey Lasry, Adar Zinger, Moshe Biton, Eli Pikarsky, Asaf Hellman, Atul J. Butte, Yinon Ben-Neriah

**Affiliations:** Institute for Computational Health Sciences, University of California, San Francisco, California 94158 USA; The Lautenberg Center for Immunology and Cancer Research, IMRIC, Hebrew University—Hadassah Medical School, Jerusalem, 91120 Israel; Developmental Biology and Cancer Research, IMRIC, Hebrew University—Hadassah Medical School, Jerusalem, Israel

**Keywords:** Inflammation, Parainflammation, p53 mutations, NSAID treatment, Cancer prevention, Genomics

## Abstract

**Background:**

Chronic inflammation has been recognized as one of the hallmarks of cancer. We recently showed that parainflammation, a unique variant of inflammation between homeostasis and chronic inflammation, strongly promotes mouse gut tumorigenesis upon p53 loss. Here we explore the prevalence of parainflammation in human cancer and determine its relationship to certain molecular and clinical parameters affecting treatment and prognosis.

**Results:**

We generated a transcriptome signature to identify parainflammation in many primary human tumors and carcinoma cell lines as distinct from their normal tissue counterparts and the tumor microenvironment and show that parainflammation-positive tumors are enriched for p53 mutations and associated with poor prognosis. Non-steroidal anti-inflammatory drug (NSAID) treatment suppresses parainflammation in both murine and human cancers, possibly explaining a protective effect of NSAIDs against cancer.

**Conclusions:**

We conclude that parainflammation, a low-grade form of inflammation, is widely prevalent in human cancer, particularly in cancer types commonly harboring p53 mutations. Our data suggest that parainflammation may be a driver for p53 mutagenesis and a guide for cancer prevention by NSAID treatment.

**Electronic supplementary material:**

The online version of this article (doi:10.1186/s13059-016-0995-z) contains supplementary material, which is available to authorized users.

## Background

Inflammation is one of the enabling hallmarks of cancer [1] and it has been estimated that approximately 20 % of cancers are caused by chronic inflammation [2, 3]. Tumor-promoting inflammation can contribute to various stages of tumor development, from tumor initiation to metastasis [4]. We recently developed and characterized a mouse model of intestinal cancer based on tissue-specific ablation of *CKIα* [5, 6]. Inducible ablation of *CKIα* in the gut epithelium has several immediate consequences, Wnt activation due to stabilization of β-catenin, induction of DNA damage response with robust p53 activation, and elicitation of a low-grade inflammatory response in the epithelium. This inflammatory reaction, which is detected by mRNA and protein analysis of the gut epithelial cells, is an atypical one, having a relatively low secretory component and, conspicuously, is not accompanied by a typical inflammatory cell infiltrate in *CKIα*-deficient mucosa. We coined this low-grade inflammatory reaction “parainflammation” (PI) based on its relationship to a term defined by Medzhitov: a low-grade inflammatory response at an intermediate state between tissue homeostasis and classic inflammation which can be induced by persistent tissue stress, serving to restore tissue homeostasis [7]. It has been proposed that PI could play a role in several conditions, such as aging and obesity [8]. In contrast to “classic” inflammation, often ignited by an extrinsic assault such as bacterial infection, PI may erupt due to tissue-intrinsic assaults, such as DNA damage [6, 7], and cooperate with the tumor suppressor *p53*, contributing to tissue protective senescence and counteracting tumor progression. Upon p53 ablation, however, PI loses its beneficial role and, instead, contributes to carcinogenesis [6]. Here we constructed a PI gene signature based on analysis of the *CKIα* and *CKIα-p53*-deficient gut epithelium and, using existing databases, examined this signature and its implications in thousands of human tumors and cell lines. We noticed a striking occurrence of PI in several cancer types distinct from their normal tissue counterparts. Our study indicates that PI is an important factor in tumor development, with a significant influence on prognosis. Notably, we found that PI can be markedly attenuated in human samples by non-steroidal anti-inflammatory drug (NSAID) treatment, possibly alluding to a mechanism of cancer prevention.

## Results

### Deriving the PI gene signature from *CKIα*-deficient mice

**Fig. 1:**
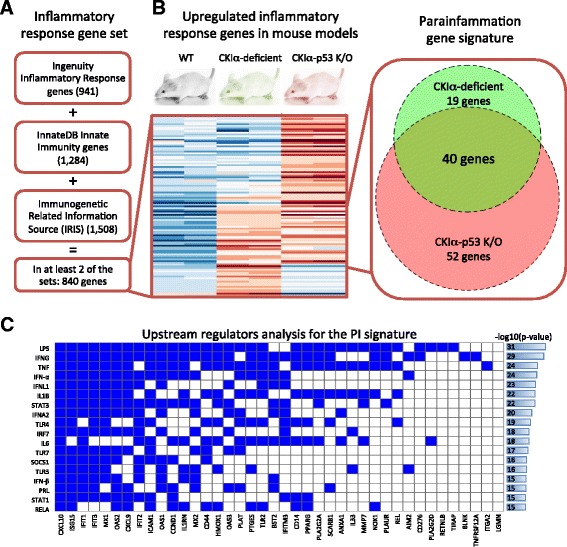
The parainflammation signature. **a** An inflammatory response gene set was generated by combining genes that are found in at least two of three manually curated databases: the Ingenuity inflammatory response gene list, InnateDB innate immunity genes, and the Immunogenetic Related Information Source (IRIS). **b** Differential expression analysis of *CKIα*-deficient and *CKIα-p53*-deficient mice against wild type (*WT*) revealed 59 and 92 upregulated inflammatory response genes in the single and double knock outs (*K/O*), respectively, and 40 genes upregulated in both. The 40 genes were designated as the parainflammation (*PI*) gene signature. The heatmap shows the expression levels of the 111 upregulated genes (*red*, high expression; *blue*, low expression). **c** Upstream regulatory analysis revealed a strong enrichment for genes regulated by lipopolysaccharide and immune pathways that are activated by it. The *rows* show the top enriched, non-chemical regulators, the *columns* are the 40 PI genes, and a *blue cell* represents regulation

Our strategy to derive a gene expression signature for PI consisted of two steps. First, we compiled a list of 840 inflammatory response genes. This list is based on three manually curated databases: the Ingenuity inflammatory response gene list (http://www.qiagen.com/ingenuity), InnateDB innate immunity genes [9], and the Immunogenetic Related Information Source (IRIS) [10] (Fig. 1a; Additional file 1). We then intersected the 840 genes with a list of genes found to be significantly upregulated in RNA-seq expression profiles of two mouse models featuring gut PI: *CKIα*-deficient and *CKIα-p53*-deficient gut epithelium (Additional file 2) [6]. This procedure generated a list of 40 inflammatory context genes which are upregulated in the PI mouse models, possibly representing a gain-of-function mechanism (Fig. 1b; Additional file 3: Table S1). Using Ingenuity’s upstream regulator analysis [11], we revealed a strong resemblance of the PI signature to the lipopolysaccharide (LPS) response (Fig. 1c; Additional file 4). This response is mediated via two major arms, the NF-κB and the interferon (IFN) signaling pathways [12], only the latter of which is found to be strongly associated with PI. The Ingenuity analysis also confirmed our observation of upregulation of the genes: 37 of the genes are known to be upregulated during the LPS/IFN response (based on manual curation of the literature). Nevertheless, no common regulator could be found for all PI genes, suggesting that PI is a unique inflammatory response whose origin and mechanism have yet to be described. It should be pointed out that while this signature includes innate immunity genes which are commonly expressed by leukocytes, in the mouse models these genes are explicitly expressed by epithelial cells [6]. Notably, the signature has a remarkable paucity of chemokines or other secreted factors, suggesting that PI is primarily cell-autonomous in the epithelial tissue and explaining why it doesn’t tend to initiate recruitment of immune cells.

### PI is suppressed by NSAID treatment in mouse tumors

**Fig. 2:**
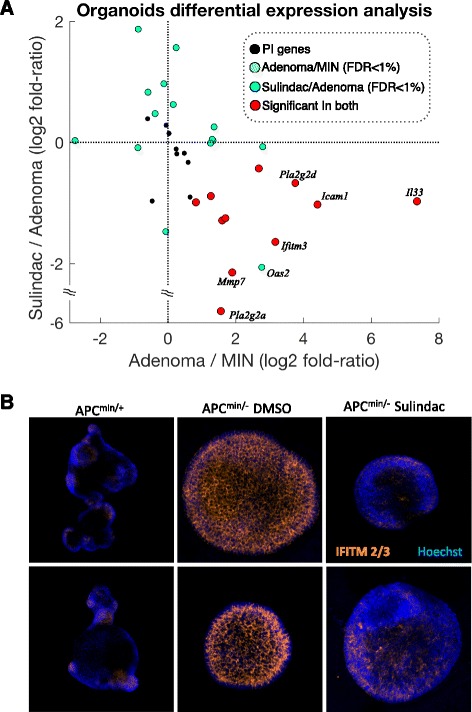
Parainflammation decreases in response to NSAID treatment in mouse organoids. **a** The adenoma/MIN log2 expression fold ratio of PI genes (*x-axis*) against the sulindac-treated adenomas/adenoma log2 expression fold ratio of PI genes (*y-axis*). *Colors* represent significance in the differential expression analyses (false discovery rate (*FDR*) <1 %). Along the *x-axis* it can be observed that 17 of 19 significant genes in adenoma/MIN are upregulated in the adenomas. Of those, 11 are significantly downregulated in the sulindac-treated samples and none are significantly upregulated. **b** *IFITM2/3* immunofluorescence in APC^min/+^, adenoma and Sulindac-treated adenoma organoids. Both rows show different fields of same experiment.

We next sought to validate the 40-gene signature for PI in an established model system. PI is associated with cellular senescence and is apparent in a pure primary epithelial tissue, such as gut epithelial organoids [6, 13]. APC-mutated human and mouse polyps represent early neoplastic lesions which mostly do not progress, possibly due to senescence-associated PI [14], thus representing a good source for testing the PI signature we characterized. We prepared organoid cultures from APC-mutated normal gut epithelium (MIN) and adenomatous polyps of APC^min/+^ mice (adenoma) and analyzed them via RNA-seq (Additional file 5). Our analysis confirmed the overexpression of many of the PI genes in adenomatous polyps in comparison with organoids prepared from the adjacent normal tissue of APC^min/+^ mouse tumors (MIN): 17 (42.5 %) of the PI genes showed significant upregulation in the tumor-derived organoids (false discovery rate < 1 %) compared with 17.5 % of all genes and 17.0 % of all inflammatory response genes (chi-square test *p* value = 4.9e-5) (Fig. 2a). Only two genes (5.0 %) showed significant, yet modest, downregulation. Quantitative PCR (qPCR) analysis confirmed these findings (Additional file 3: Figure S1).

It is not yet known why NSAID treatment has an impact on cancer prevalence, progression, and mortality in human solid tumors [15–18]. We have previously shown that gut epithelium PI can be suppressed by NSAID treatment in the *CKIα*-deficient mouse model [6]. We therefore hypothesized that cancer cells may harbor tumor-promoting PI subject to suppression by NSAID treatment. To test this hypothesis, we generated a tumor model for examining NSAID treatment effects. We prepared organoids from normal gut and adenomatous polyps of APC^min/+^ mice, treated them with the NSAID sulindac, and performed RNA-seq on three biological replicates (Additional file 6). Strikingly, of the 17 upregulated PI genes observed in the tumor organoids, 11 (64.7 %) were significantly downregulated in the sulindac-treated samples, compared with 33.2 % of all genes and 38.3 % of all inflammatory response genes (*p* value = 0.0053) (Fig. 2a). Immunofluorescence staining shows that the protein expression of PI gene *Ifitm2*/*3* (Fig. 2b) is markedly elevated in the tumor organoids and is mostly suppressed by sulindac treatment. These data validate the PI signature in a new mouse tumor model, underscoring both the epithelial origin and the tumor specificity of this new signature, and show that NSAID treatment can reduce PI.

Analogously to the procedure described above, we also found 75 inflammatory context genes which are downregulated in the PI mouse models (Additional file 3: Table S2). This list of genes is again highly enriched with LPS and IFN γ response genes but also contains interleukin (IL)2/IL4 response genes. However, we didn’t observe expression changes of those 75 genes in the organoids model, suggesting that the downregulated genes are not part of the general PI mechanism. Therefore, while it is well established that activation of innate inflammation pathways leads to both up- and downregulation of genes involved in activation and inhibition of this highly regulated network [19–21], we chose to derive the PI signature only from the upregulated genes in the mouse and organoid PI models.

### Human cancers display the PI signature discovered in mice

**Fig. 3:**
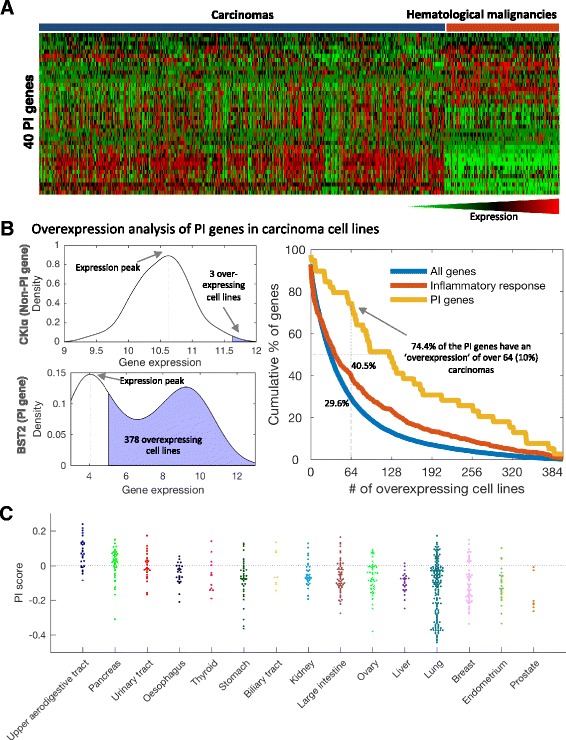
Parainflammation genes are overexpressed in carcinoma cell lines. **a** Heatmap of the expression of 39 PI genes in 634 carcinoma cell lines and 180 hematopoietic and lymphoid cancer cell lines from CCLE. One PI gene, *IFITM3*, is not represented in CCLE. Of the PI genes, 19 show significantly higher expression in carcinomas compared with cancers originated from immune cell types; 10 PI genes are more abundant in immune cancers. **b** *Left*: the distribution of expression of two representative genes across 634 carcinoma cell lines from CCLE. We detected the expression peak and counted the number of samples that express the gene twofold (1 in log2 scale) more than the peak. The top example, the gene *CSNK1A1* (CKIα), which here represents a housekeeping gene, is a typical normally distributed gene with low “overexpression” rate and the bottom example, *BST2*, which is part of the PI signature, shows a gene with a bimodal expression pattern, corresponding to a high “overexpression” rate. *Right*: the cumulative overexpression rates for the PI genes, all inflammatory response genes, and all genes. Of the PI signature genes, 29 (74.4 %) are overexpressed in at least 10 % of the carcinoma cell lines (≥64 samples) compared with 29.6 % and 40.5 % of all and inflammatory response genes, respectively. The *yellow curve* (PI genes) shows remarkably higher levels of overexpression along the whole graph. **c** Spread plot of tThe PI score in 634 carcinoma cell lines grouped by tissue types. The *dashed blue line* differentiates PI+ and PI− samples as defined by the proportion of PI+ of tumor samples

Observing PI in mouse tumors prompted us to look for a similar phenomenon in human cancers. To that end, we first analyzed the expression of 40 human homologs of the mouse PI genes in human cancers, utilizing data from the Cancer Cell Line Encyclopedia (CCLE) [22]. Whereas immune and inflammatory genes are normally expressed in hematopoietic cells, a wide range of carcinoma cell lines (*n* = 634) expressed the PI genes to a higher level than hematopoietic and lymphoid cancer cell lines (*n* = 180) (Fig. 3a; Additional file 3: Figure S2). Further investigation revealed that, compared with non-PI genes, many PI genes are broadly overexpressed in a subset of the cell lines. To quantify the distribution of PI genes in cell lines, we computed the expression distribution in all the carcinoma cell lines and identified the peak of the distribution. Then, by defining overexpression as twofold expression level over the peak (1 in log2 expression levels), we counted the number of cell lines overexpressing each gene (examples in Fig. 3b). Our analysis revealed that 74.4 % of the PI genes are overexpressed in more than 10 % of the carcinomas, significantly more than a non-discriminatory pool of random inflammatory response genes (40.5 %, chi-square test *p* value = 2.9e-5) and all genes (29.6 %, *p* value = 1.0e-9) (Fig. 3b). The median number of overexpressing cell lines was 125 (19.7 % of carcinoma samples) for the PI genes compared with 39 (6.2 %, U-test *p* value = 2.7e-5) across random inflammatory response genes and only 31 (4.9 %, *p* value = 2.6e-8) across random genes.

We next computed a score for each cell line by performing single-sample gene set enrichment analysis (ssGSEA) [23] for the PI gene signature. We defined this score, which is an enrichment measure of the overexpression of the PI genes, as the PI score (Additional file 7). The PI score revealed major differences in PI both between cancer types and within cancer types, varying from high levels of PI in head and neck and pancreatic cancer to low levels in samples originating from prostate and liver (Fig. 3c). Thus, the PI expression and PI score patterns suggest that PI occurs across most cancer types, although not uniformly. It is important to note that while the PI score is based on a signature of 40 genes, the PI phenomenon is not restricted to these genes alone but has a wide effect on numerous genes over many different molecular functions (Additional file 8). Finally, we observed a high correlation between the PI score and scores derived from the downregulated PI genes (Spearman R = 0.654, *p* value <1e-20; Additional file 3: Figure S3). This once again confirms the validity of our gene sets in human and the relevance of the PI mouse models to a phenomenon that is also observed in human.

Next, we explored the representation of the PI signature in primary human cancers. As differences in PI expression between individual samples may be explained solely by differences in purity levels of the samples [24], we designed a simple adjustment procedure for removing inflammatory gene expression originating from immune infiltrations (Additional file 3: Figure S4). This adjustment procedure consists of two steps: first, utilizing expression data of normal tissues from the Genotype-Tissue Expression (GTEx) project [25], we learned the normal association of gene expression with immune infiltrations in each tissue type. We then used the expression level of *PTPRC* (*CD45*), a pan-hematopoietic exclusive marker expressed in all leukocyte cell types but not in other tissues, as an estimate for immune infiltrations. Finally, we applied the gene-specific, tissue-specific learned slopes to adjust the expression levels of The Cancer Genome Atlas (TCGA) tumor samples. This procedure diminishes expression differences among samples which are most likely explained by differences in purity.

**Fig. 4:**
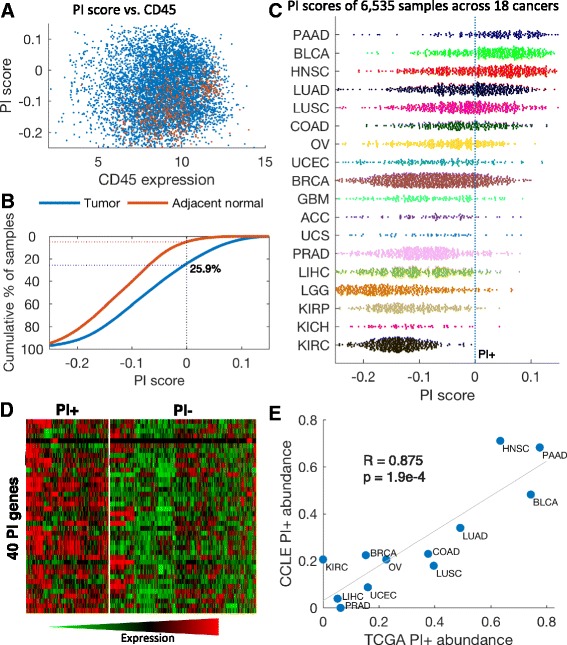
Pan-cancer parainflammation in human cancers. **a** PI scores (*y-axis*) of all 6535 tumor samples (*blue*) and 582 adjacent normal samples (*red*) versus the CD45 expression level (*x-axis*). No correlation is observed between the PI score and CD45 after the adjustment. **b** PI scores in the tumor samples (*blue*) and the adjacent normal samples (*red*). The y-axis is the cumulative percentage of samples over the score in x; 25.9 % of the tumor samples (*dashed blue line*) are over a threshold which only 5 % of the adjacent normal tissues pass (*dashed red line*). The PI score is shifted accordingly, so PI+ samples have positive scores. **c** Spread plot of the PI score in 6535 tumor samples from 18 cancer types. The *dashed blue line* differentiates PI+ and PI− samples. *PAAD* pancreatic adenocarcinoma, *BLCA* bladder carcinoma, *HNSC* head and neck squamous cell carcinoma, *LUAD* lung adenocarcinoma, *LUSC* lung squamous cell carcinoma, *COAD* colon adenocarcinoma, *OV* ovarian serous cystadenocarcinoma, *UCEC* uterine corpus endometrial carcinoma, *BRCA* breast carcinoma, *GBM* glioblastoma multiforme, *ACC* adrenocortical carcinoma, *UCS* uterine carcinosarcoma, *PRAD* prostate adenocarcinoma, *LIHC* liver hepatocellular carcinoma, *LGG* lower grade glioma, *KIRP* kidney renal papillary cell carcinoma, *KICH* kidney chromophobe, *KIRC* kidney renal clear cell carcinoma. **d** Heatmap of expression profiles of PI genes across TCGA samples: *left*, PI+ samples; *right*, PI− samples. Different subsets of PI genes are expressed in PI+ samples. The expression levels presented are after adjustment to immune infiltrations and are standardized across all samples. **e** Correlation of the fraction of PI+ samples in each tumor type from TCGA with corresponding tissue origin in CCLE. The same types of cancers have low or high PI+ levels in tumors and in cell lines. The Pearson coefficient is presented

We collected gene expression data for 6523 primary tumors and 582 patient-matched normal samples of 18 cancer types from TCGA (Additional file 3: Table S3; http://cancergenome.nih.gov) and performed the adjustment procedure. As in the cancer cell lines, we computed the scores for the PI gene signatures using the adjusted expression of each tumor sample (Additional file 9). Cancer samples demonstrated a wide range of PI scores regardless of *CD45* expression (Fig. 4a; Additional file 3: Figure S5). Furthermore, malignant tumors demonstrated significantly higher levels than adjacent normal samples, both overall and across most tested cancer types (Fig. 4b; Additional file 3: Figure S6). As in cell lines, the PI score analyses revealed considerable differences between cancer types and within cancer types (Fig. 4c). We next defined the PI threshold as the score that appears in only 5 % of the adjacent normal samples; cancer samples over the threshold were designated as PI positive (PI+). Strikingly, over all cancer types 25.9 % of the tumor samples were PI+, compared with a null expectation of 5 %, with varying proportions among cancer types, from 77.7 % in pancreatic adenocarcinoma (PAAD) to none in kidney renal clear cell carcinoma (KIRC). The PI score of a sample is highly correlated with the number of upregulated genes in the sample (R = 0.649; Additional file 3: Figure S7). Accordingly, the median number of PI genes activated in PI+ samples is 17 (42.5 %; compared with eight in PI− samples), the same number we observed to be upregulated in the adenoma organoids. Interestingly, we observed that different sets of PI genes are activated in different cancer types (Fig. 4d). Finally, using the 25.9 % proportion of PI+ samples, we determined a threshold of PI+ samples in the cancer cell lines dataset (where there are no normal samples to determine the threshold). Remarkably, we found high concordance in the abundance of PI+ samples in cancer types between tumors and cell lines (Pearson coefficient R = 0.875, *p* value = 1.9e-4 (Fig. 4e). These results again suggest that the PI gene signature is being expressed by the cancer cells, distinct from immune infiltration.

**Fig. 5:**
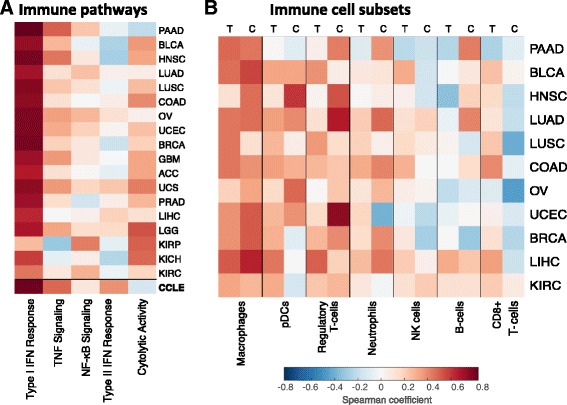
Cancer parainflammation resembles macrophage infiltration. **a** Heatmap of the Spearman correlations between the PI score and immune functional gene sets across different cancer types. Correlations in CCLE are shown as well (Additional file 3: Tables S5 and S6). **b** Heatmap of the Spearman correlations between the PI score and the immune subset enrichments calculated using gene sets (Additional files 6 and 9) across different cancer types derived from both TCGA (*T*) and CCLE (*C*). Similar correlation trends are observed for a cancer type whether the data were derived from TCGA or CCLE, suggesting that the correlation is due not to association of PI with immune subset presence but to shared functionality with the gene signatures. *PAAD* pancreatic adenocarcinoma, *BLCA* bladder carcinoma, *HNSC* head and neck squamous cell carcinoma, *LUAD* lung adenocarcinoma, *LUSC* lung squamous cell carcinoma, *COAD* colon adenocarcinoma, *OV* ovarian serous cystadenocarcinoma, *UCEC* uterine corpus endometrial carcinoma, *BRCA* breast carcinoma, *GBM* glioblastoma multiforme, *ACC* adrenocortical carcinoma, *UCS* uterine carcinosarcoma, *PRAD* prostate adenocarcinoma, *LIHC* liver hepatocellular carcinoma, *LGG* lower grade glioma, *KIRP* kidney renal papillary cell carcinoma, *KICH* kidney chromophobe, *KIRC* kidney renal clear cell carcinoma

We noted earlier that the PI gene signature is enriched in genes related to the IFN signaling pathway but not to the NF-κB pathway. To test this hypothesis further we first expanded the list of PI genes, identifying 215 genes with expression levels highly correlated with the PI score (R > 0.5) in the carcinoma cell lines (Additional file 8). We then performed transcription factor binding enrichment analysis using the ENCODE ChIP-Seq Significance Tool [26] (Additional file 3: Table S4). The analysis again affirmed our claim that PI has a distinct pattern of inflammation, where the top enriched transcription factors were STAT2, IRF1, STAT1 and STAT3, c-Fos, and PRDM1, but only modest enrichment for a key regulator of inflammation such as NF-κB. We further utilized enrichment scores of functional immune gene sets (Additional file 3: Table S5) and correlated them with the PI score in both tumors and cell lines. As expected, this analysis revealed high correlations in all datasets with the type I IFN response, which is a hallmark of PI, but a much weaker association with the tumor necrosis factor and NF-κB signaling pathways (Fig. 5a; Additional file 3: Table S6). We also did not detect any correlation with the immune cytolytic activity metric [27], which is a well-described anti-tumor immunity measure. It should be pointed out that the same correlations were also found in the CCLE dataset, again showing that PI is activated in the tumor and not in its microenvironment. These results again support our claim that PI is distinct from canonical chronic inflammation or other previously described inflammatory responses.

Notably, certain PI genes are members of the Toll-like receptor (TLR) activation pathway (TLR2, CD14, and TIRAP) and, when upregulated, could have mediated an innate immune response to tissue-associated microbiota, igniting conventional inflammation with inflammatory infiltrate, secondary to PI. We therefore hypothesized that PI may enhance the recruitment of certain immune cell subsets to the tumors. To this end, we utilized hematoxylin and eosin (H&E) estimations provided by TCGA and gene signature enrichments of immune subset types from Rooney et al. [27] (Additional file 3: Table S5) and associated them with PI scores in tumor samples across cancer types. H&E estimations of major immune subtypes (lymphocytes, monocytes, and neutrophils) did not, however, show significant associations with PI scores across different cancer types (Additional file 3: Table S7). We further correlated PI scores of individual tumors with specific immune subsets based on gene signature enrichments in both TCGA and CCLE samples (Fig. 5b; Additional file 3: Table S8). Among the immune subsets the PI score demonstrated highest correlations across tumor types with the macrophage signature (average Spearman coefficient = 0.362). However, we observed the same trend of correlation between the PI score and macrophages in cell lines (average R = 0.407). This observation rules out the possibility that PI is dependent on macrophages infiltrating the tumor. Moreover, whereas the role of macrophages in orchestrating PI has been previously suggested [7], our finding supports this inference yet suggests that the tumors themselves may fulfill the macrophage inflammatory function in PI by expressing macrophage-relevant genes. Importantly, we did not observe any correlation with CD8+ enrichment, which is the main component of the “immunoscore” [28], thus suggesting that PI may represent a different immunotype, which, similarly to the immunoscore, may serve as a clinical parameter in evaluating tumorigenesis.

Thus, the PI signature is widely expressed in human tumors, distinguishing the tumor cells from adjacent normal tissue and the tumor microenvironment, with certain cancer types having stronger PI signatures than others. PI appears to be primarily a cancer cell-autonomous phenomenon, distinct from all other well-established cancer-promoting immune inflammatory responses.

### PI is associated with poor prognosis and p53 mutations

**Fig. 6:**
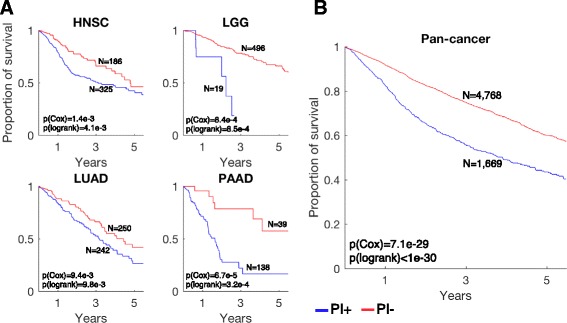
Parainflammation is associated with worse prognosis. **a** Kaplan-Meier plots for PI+ samples (*blue*) versus PI− samples (*red*) in head and neck squamous cell carcinoma (*HNSC*), lower grade glioma (*LGG*), lung adenocarcinoma (*LUAD*), and pancreatic adenocarcinoma (*PAAD*). The *p* values were calculated using Cox proportional hazard regression of the PI scores and the log-rank method between PI+ and PI− samples. **b** Pan-cancer survival analysis of 6437 patients with survival data and PI scores

Based on the analysis of the *CKIα*-deficient mouse models, PI itself may either serve as a tumor suppressor mechanism or help promote carcinogenesis [6]. To determine PI’s role in human cancer, we analyzed clinical data from TCGA, which allowed us to examine the association of PI with survival time and other clinical features available from TCGA database in 18 cancer types. Our analysis revealed higher mortality rates for patients with high PI scores in most cancer types (Additional file 3: Table S9). Prominent examples of high PI score tumors associated with bad prognosis are head and neck squamous cell carcinoma (HNCS; Cox regression *p* value = 1.4e-3), low-grade glioma (LGG; *p* value = 8.4e-4), lung adenocarcinoma (LUAD; *p* value = 9.4e-3), and pancreatic adenocarcinoma (PAAD; *p* value = 6.7e-5). Median time of mortality of PI+ compared with PI− patients was 1.54-, 3.50-, 1.33-, and 3.53-fold faster, respectively (Fig. 6a; Kaplan-Meier plots). A pan-cancer survival analysis of all samples confirmed a negative correlation of PI with survival, showing a consistent earlier mortality for the PI+ patients (*p* value = 7.1e-29; Fig. 6b). Notably, low-grade glioma has a low overall PI score; however the small subset of PI+ patients have a significantly lower survival rate, indicating a value of screening for PI even in tumor types with low PI. Analysis of other clinical features, including age, gender, race, and smoking, showed that the PI score is generally independent of these variables (Additional file 3: Table S10). Accordingly, multivariate regression analysis controlling for age and smoking did not change the power of the PI score in predicting survival (Additional file 3: Table S9).

**Fig. 7:**
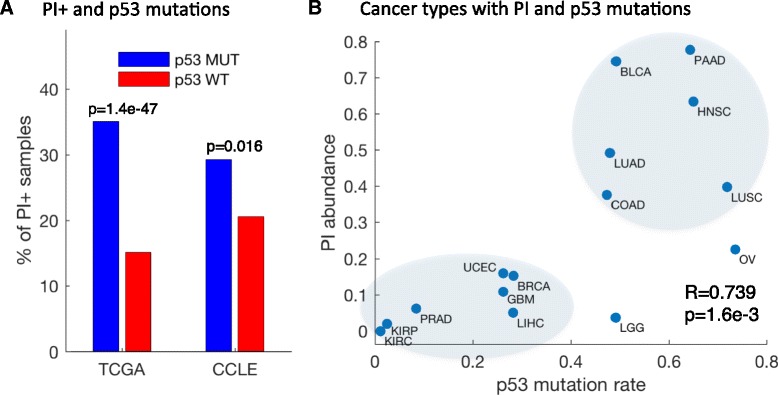
Parainflammation is associated with p53 mutations. **a** Percentage of p53 nonsense mutated PI+ samples (*blue*) versus percentage of p53 WT PI+ samples (*red*) in TCGA and in CCLE. **b** Abundance of p53 missense mutations (*x-axis*) versus the abundance of PI+ samples (*y-axis*) in 15 cancer types with sufficient information. Cancer types with higher rates of p53 nonsense mutations tend to have higher rates of PI. The Spearman coefficient is presented

In the *CKIα*-deficient mouse model, PI promotes carcinogenesis only after loss of *p53*, suggesting that PI+ samples will be enriched with *p53* mutations. We therefore investigated the relationship of PI and the status of p53 in human cancer. By analyzing the mutation data from whole-exome sequencing provided by TCGA across all samples with expression data, we found 1309 samples with mutations in the *TP53* gene versus 2363 with wild-type (WT) *TP53*. The PI score was significantly higher in the mutated p53 samples (U-test *p* value = 1.4e-47) and 35.4 % of the p53 mutated samples were PI+ compared with 15.2 % in WT *p53* samples (Fig. 7a). This result was recapitulated in the carcinoma cell lines—29.3 % PI+ in mutated p53 and 20.6 % in WT—although less significant (*p* value = 0.016). p53 mutations are observed more frequently in cell lines than in primary tumors (59.8 % versus 35.7 %), possibly reflecting the growth advantage of p53 mutants in tissue culture, which may hinder a stronger association between p53 mutations and PI in cancer cell lines. Notably, cancer types with known, albeit unexplained low rates of *p53* mutations, such as kidney cancer types and prostate adenocarcinoma (PRAD) [29], tend to show low rates of PI, whereas cancers with high *p53* mutation rates, such as pancreatic adenocarcinoma (PAAD) and colon adenocarcinoma (COAD), show high rates of PI (R = 0.740, *p* value = 0.002) (Fig. 7b). Finally, it is well known that patients harboring tumors with p53 mutations have significantly worse prognosis [30] (in TCGA: Cox regression *p* value = 4.9e-15). We tested whether the association of PI with survival relates to p53 mutations. For 2954 patients with both mutations and clinical data, our analysis revealed only a small decrease in significance between controlled and uncontrolled Cox regression analyses (*p* value without controlling for p53 = 4.7e-17, *p* value with controlling for p53 = 2.4e-11), indicating that the poor survival associated with PI cannot entirely be attributed to p53 loss.

### PI response to NSAID treatment in human tumors

**Fig. 8:**
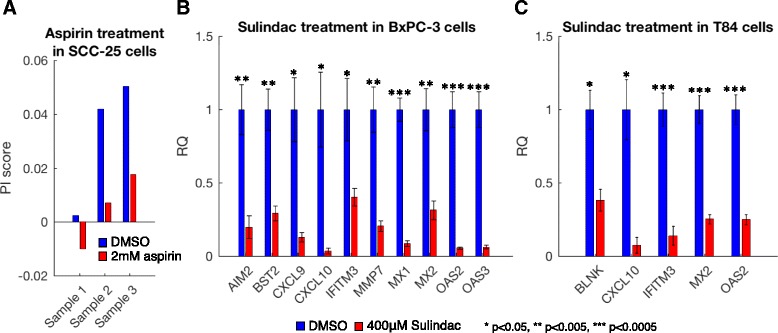
Parainflammation decreases in response to NSAID treatment in human cell lines. **a** average of the differential expression (in log2 scale) of three replicates each of control and aspirin-treated SCC-25 cells for the PI genes. A kernel smoothed regression curve for all genes is presented as reference (*red*). The *p* value presented was calculated using a Wilcoxon rank-sum test. **b** qPCR analysis of highly expressed PI genes in control and sulindac-treated BxPC3 cells. *Error bars* show the standard error of the mean (SEM) of triplicates. **c** qPCR analysis of highly expressed PI genes in control and sulindac-treated T84 cells. *Error bars* show the SEM of triplicates

Our mouse tumor data indicate that tumor PI is suppressed by sulindac treatment (Fig. 2). Prolonged NSAID treatment was found to reduce both the incidence and the mortality of human cancer [15–17]. To study the relevance of PI in NSAID treatment in humans, we first searched for public expression data of NSAID treatment in cancer cell lines. While there are several such databases, notably the Broad Institute Connectivity Map (C-Map) [31], most of the data are limited to cell lines that show low PI levels before treatment and, therefore, no treatment effect is expected. Nevertheless, we observed one particular cell line with a high PI score, SCC-25, an oral cancer cell line treated with 2 mM of aspirin (GSE58162). This cell line has a high PI score, which ranks it 19th out of 634 CCLE carcinomas. We observed a sharp decrease in the expression of the PI genes in response to the treatment, and in two of the three replicates the PI gene set is significantly downregulated compared with all genes: U-test *p* value = 8.6e-4, 0.318, and 0.009 in the three replicates, respectively. Similarly, we found that the PI score decreased in all three replicates following aspirin treatment (Fig. 8a). We further noticed that the response to aspirin is significantly enhanced when examining a large set of genes that are upregulated in correlation with the PI score; 74.0 % of the top 215 correlated genes are downregulated in response to aspirin treatment (U-test *p* value = 8.1e-25; Additional file 3: Figure S8).

To confirm these computational findings, we treated two human cancer cell lines, predicted to have high PI scores from the CCLE dataset analysis, with sulindac: the BxPC3 cell line, a pancreatic cancer cell line with a PI score which ranks it ninth out of the 634 CCLE carcinomas, and the T84 cell line, a colorectal cancer cell line ranked 18th. Sulindac treatment led to a decrease of more than 50 % in the expression of all tested PI genes which are highly expressed in BxPC3 and T84 cells, respectively (Fig. 8b, c). This, together with the analyses of the SCC-25 cell line and the mouse tumor organoids (Fig. 2), confirms the repressive effect of NSAID treatment on PI, suggesting that part of the cancer-prevention mechanism of NSAIDs may be attributed to PI suppression.

## Discussion

Inflammation is emerging as one of the hallmarks of cancer [1] yet histologically detectable chronic inflammation characterizes early tumor development in only a minority of solid tumors [32]. In this respect it is surprising that NSAID treatment is remarkably effective in reducing mortality rates associated with major human solid tumors, albeit their mechanism of action in cancer is controversial and there is no common property distinguishing cancers in which NSAID treatment is beneficial. NSAIDs are non-selective Cox2 inhibitors and it is thus not surprising that they are effective in cancers where Cox2 is indeed elevated. However, NSAIDs are also effective in cancers where Cox2 is not activated, occurring through hereditary or stochastic mutations and not preceded by prolonged inflammation. This indicates that inflammation may have a covert course that plays a major unappreciated role in cancer. Accordingly, in spite of many studies addressing this question, the mechanism of NSAID action in cancer prevention remains mostly elusive. Medzhitov coined the term parainflammation for a low-grade inflammatory response, referring to an adaptive response due to tissue stress or malfunction [7]. Here we defined PI on the basis of analysis of existing databases and experimental data: an epithelial-autonomous inflammatory response observed in genetically modified mouse models. We then investigated the occurrence of PI in human cancer, asking (a) whether PI is a universal cancer phenomenon and (b) does PI have clinical/prognostic implications in human cancer. Using several large sets of human cancer samples, we detected frequent occurrence of PI in human cancers with several interesting features: PI is cancer cell-autonomous, readily detectable in human carcinoma cell lines; its repertoire is distinct from other common inflammatory signatures; and it doesn’t bear the phenotypic hallmarks nor some of the regulatory characteristics of common inflammatory reactions (e.g., NF-κB). PI is very common in certain types of cancer, including bladder, head and neck, cervical, and colorectal cancer, and conspicuously absent in other types, such as hepatocellular carcinoma, prostate and endometrial adenocarcinoma, and low grade glioma.

In the *CKIα*-deficient mouse model, PI promotes tumorigenesis only after loss of p53. We therefore investigated whether p53 loss/mutation in human tumors is associated with occurrence of PI. Indeed, the p53 status of the tumor displays a high association with PI in a variety of cancer types. A similar association of p53 mutation or loss with PI occurs across the entire spectrum of cancer types: those with low PI, like prostate, liver, thyroid, and melanoma, have few p53 mutations. Why certain cancer types have low p53 mutation frequencies with no evidence of p53 pathway suppression is an enigma. The strong association between PI and p53 mutations in cancer suggests that PI may be one of the major driving forces for inactivating the p53 pathway. This corresponds to the relationship between PI and p53 deficiency in mouse tumor models, showing a sharp PI switch from a tumor suppressor to a tumor promoter mechanism upon p53 loss [6]. Such a tumorigenesis switch mechanism may be a particularly powerful cancer driver mechanism and is thus possibly one of the main mechanisms enforcing p53 mutations in cancer, which cannot be substituted by loss of other tumor suppressor genes. While we are not aware yet of other means of switching PI from a cancer suppressor to a promoter, it is possible that certain other tumor-specific genetic aberrations may fulfill a function similar to p53 inactivation, pushing cancer progression. Indeed, PI is associated with worse prognosis, beyond the p53 status of the tumor. These retrospective analyses could indicate the value of screening individual tumors for PI manifestation as a prognostic or treatment/prevention tool. Particularly relevant in this aspect is NSAID treatment. Retrospective analysis of cancer patients who were routinely treated with aspirin, including low dose aspirin, for non-cancer indications showed a surprisingly beneficial effect in reducing cancer mortality following surgical removal of the tumor [17]. Nearly all the cancer types in which beneficial aspirin effects were noted (e.g., colorectal, pancreatic, lung, and breast) [17, 18, 33] are characterized by high PI, either throughout the entire cancer type or a significant subtype, calling for implementation of PI screening of tumor biopsy or resection samples. Supporting our hypothesis implicating PI suppression as an important anti-tumorigenic mechanism of NSAID is our study of a mouse tumor model and human cancer cell lines displaying high PI: we demonstrated effective sulindac repression of the majority of the tested PI genes upregulated in the tumor cells. Thus, whereas we are still far from understanding the origin and mechanisms of PI emergence in tumors, its recognition and monitoring may be of great value in the clinical care of cancer.

## Conclusions

In this work, we have characterized a novel PI signature present in 25.9 % of all human tumors and in the vast majority of certain types of cancer, such as gastrointestinal, lung, bladder, and head and neck tumors. PI has a distinct signature, originating endogenously from the tumor cells, and does not coincide with canonical inflammation. Our data indicate that PI is linked to p53 mutations, suggesting it might be a major driving force for p53 mutation. As PI is suppressible by NSAID treatment in vitro and is particularly prominent in cancers in which aspirin treatment is beneficial, we propose that a tumor PI signature should be tested as a potential biomarker/clinical indication for NSAID chemoprevention and treatment of cancer.

## Methods

### Data analysis

#### PI gene signature

We obtained raw RNA-seq counts from Pribluda et al. [6] for WT, *CKIα*-deficient and *CKIα-p53* doubly deficient gut epithelium, each with two replicates (Additional file 2). Using the DESeq2 package for R [34], we calculated *p* values and fold ratio changes against the WT counts. Upregulated genes were chosen as genes with fold ratio >2 and adjusted *p* value <0.01. Genes upregulated in the mouse models were then intersected with our list of inflammatory response genes. The downregulated gene signature was defined in the same manner but was more than twofold lower in the deficient mouse models compared with WT.

#### Upstream regulator analysis

The analysis was performed through the use of QIAGEN’s Ingenuity® Pathway Analysis (IPA®, QIAGEN Redwood City, http://www.qiagen.com/ingenuity). Figure 1c shows the top 18 non-chemical regulators; Additional file 4 presents all data.

#### Adjustment for blood admixture

Infiltration of immune cells into tumor samples may influence the analysis of gene expression profiles of the tumor cells and these should therefore be adjusted. The level of blood cells in tumor samples may be efficiently analyzed using leukocyte-specific expression profiles, e.g., by the ESTIMATE score [35]. We found tight correlation between the ESTIMATE measurement and CD45 expression levels over tumor samples across all cancer types (Additional file 3: Figure S4a). The ESTIMATE score, which is calculated using a gene signature of 141 genes, might be perturbed by our notion of PI; thus, we adjusted the expression levels of each gene according to CD45 expression alone. Using expression data of normal samples from the Genotype-Tissue Expression Project (GTEx) [25], we first fit a linear curve for each gene according to its expression level and the expression level of CD45 in each tissue type (Additional file 3: Figure S4b). The slope learned from this section represents the normal tissue-specific association between the gene expression levels and the immune infiltration levels. Using this slope we then adjust the expression levels of the gene for all TCGA samples in the corresponding cancer type (Additional file 3: Figure S4c). Finally, the expression of all samples is shifted so the minimal level for each gene is 0.

#### PI score

The PI score is the ssGSEA score of the 40 PI genes (Additional file 3: Table S1). A TCGA tumor sample was considered PI positive (PI+) if its PI score is over 0.2951. This threshold is found in less than 5 % of TCGA adjacent normal samples and corresponds to 25.9 % of the tumor samples overall. In the CCLE dataset only 9.5 % of the carcinoma cell lines were over this threshold. However, the ssGSEA method is highly sensitive to different expression platforms with different numbers of genes and is thus incomparable between these. Of the carcinoma cell lines, 25.9 % have a PI score over 0.1859. This notion is true for other datasets used in this article for calculating the PI scores: the scores should only be compared between samples from the same expression-measuring platform in the species. To allow some degree of comparison between the different datasets, for visualization the scores were shifted in each data set such that PI+ samples have positive scores.

#### Organoid culture

Organoids were produced from WT and APC^min/+^ mice as previously described [13, 36]. For adenoma and APC^min/+^ organoids, adenomas and normal adjacent tissue were separated and processed accordingly. All organoids were grown in Advanced DMEM/F12 culture medium (Gibco) supplemented with Noggin, EGF, bFGF (Peprotech, 1:1000), R-spondin1 (Peprotech, 1:500) and B27 (Gibco, 1:50). For staining, organoids were fixed in 4 % paraformaldehyde and incubated overnight with the primary antibody (IFITM2/3 - 1:200, Abcam). Secondary antibody was Alexa fluor-647 donkey anti-rabbit (Molecular Probes, 1:1000). Hoechst (1 μg/ml, Molecular Probes) was used to stain nuclei.

#### NSAID treatment of human cancer cell lines

BxPC3 cells were grown in RPMI medium. T84 cells were grown in 1:1 DMEM/F12 medium. For sulindac treatment, cells were incubated with 400 μM sulindac (Sigma) for 48 h before harvesting. Controls were treated with DMSO, the NSAID vehicle.

#### RNA extraction and qPCR analysis

RNA was extracted using the miRNeasy kit (Qiagen). cDNA was produced using qScript cDNA kit (Quanta). Primers used for qPCR analysis can be found in Additional file 3: Table S11.

## Abbreviations

CCLE, Cancer Cell Line Encyclopedia; GEO, Gene Expression Omnibus; GTEx, Genotype-Tissue Expression; H&E, hematoxylin and eosin; IFN, interferon; IL, interleukin; KIRC, kidney renal clear cell carcinoma; LPS, lipopolysaccharide; NSAID, non-steroidal anti-inflammatory drug; PAAD, pancreatic adenocarcinoma; PI, parainflammation; qPCR, quantitative PCR; ssGSEA, single-sample gene set enrichment analysis; TCGA, The Cancer Genome Atlas; WT, wild type.

## Additional files

Additional file 1: List of 840 inflammatory response genes based on three manually curated databases: inflammatory response gene lists curated by Ingenuity, InnateDB innate immunity genes, and the Immunogenetic Related Information Source (IRIS). (XLSX 74 kb) Additional file 2: Results from differential expression analyses of *CKIα*-deficient and *CKIα-p53*-deficient gut epithelium versus wild-type gut epithelium. Raw RNA-seq counts were obtained from Pribluda et al. [6]. Each set has two replicates. The analysis was performed using the DESeq2 package for R. (XLSX 4406 kb) Additional file 3: Figure S1. qPCR of PI genes in organoids. **Figure S2.** Expression of PI genes in cell lines. **Figure S3.** Correlation between the PI scores derived from up- and downregulated genes. **Figure S4.** Adjusting TCGA expression levels to immune infiltrations. **Figure S5.** Correlation of the PI score with CD45 expression. **Figure S6.** PI scores in TCGA cancer and adjacent normal samples. **Figure S7.** The PI score is correlated with the number of activated PI genes. **Figure S8.** PI-associated gene responses to aspirin treatment. **Table S1.** Parainflammation gene signature. **Table S2.** Down-regulated parainflammation genes. **Table S3.** List of samples acquired from TCGA. **Table S4.** Transcription factor binding enrichment analysis of PI genes. **Table S5.** Immune gene signatures. **Table. S6.** PI score and functional immune gene sets. **Table S7.** PI score and immune subsets estimated by hematoxylin and eosin staining. **Table S8.** PI score and immune subset estimate by gene signatures. **Table S9.** Survival and parainflammation. **Table S10.** Clinical features and parainflammation. **Table S11.** Primers used for qPCR analyses. (PDF 1608 kb) Additional file 4: Results from an Ingenuity upstream regulator analysis performed on the 40 PI genes. (XLSX 123 kb) Additional file 5: Results from a differential expression analysis of organoid cultures from APC-mutated normal gut epithelium (MIN) versus adenomatous polyps of APC^min/+^ mice (Adenoma). See “Methods” for organoid culture. Each set has three biological replicates. Raw reads and counts are available at the GEO under accession GSE81836. The analysis was performed using the RSEM software package. (XLSX 773 kb) Additional file 6: Results from a differential expression analysis of organoid cultures from adenomatous polyps of APC^min/+^ mice (Adenoma) versus adenomatous polyps of APC^min/+^ mice treated with sulindac. See “Methods” for organoid culture and sulindac treatment. Each set has three biological replicates. Raw reads and counts are available at the GEO under accession GSE81836. The analysis was performed using the RSEM software package. (XLSX 731 kb) Additional file 7: Parainflammation (PI) scores for 634 carcinoma cell lines from the CCLE project. The PI score is calculated using ssGSEA for the 40 PI genes. (XLSX 69 kb) Additional file 8: Spearman coefficients of correlations between gene expression profiles and the PI scores across the carcinoma cell lines. Many genes not in part of the PI signature are highly correlated with the PI score, suggesting their role in PI. (XLSX 422 kb) Additional file 9: Parainflammation (PI) scores for 6523 primary tumors and 582 patient-matched normal samples of 18 cancer types from TCGA. The PI score is calculated using ssGSEA for the 40 PI genes using the immune-adjusted expression profiles. (XLSX 260 kb)
