# Supplementary material for: Widespread parainflammation in human cancer
Source: Genome Biol. 2016 Jul 8;17:145. doi: 10.1186/s13059-016-0995-z (PMC4937599; doi:10.1186/s13059-016-0995-z)
Supplement: Additional file 3: Figure S1. — qPCR of PI genes in organoids. Figure S2. Expression of PI genes in cell lines. Figure S3. Correlation between the PI scores derived from up- and downregulated genes. Figure S4. Adjusting TCGA expression levels to immune infiltrations. Figure S5. Correlation of the PI score with CD45 expression. Figure S6. PI scores in TCGA cancer and adjacent normal samples. Figure S7. The PI score is correlated with the number of activated PI genes. Figure S8. PI-associated gene responses to aspirin treatment. Table S1. Parainflammation gene signature. Table S2. Down-regulated parainflammation genes. Table S3. List of samples acquired from TCGA. Table S4. Transcription factor binding enrichment analysis of PI genes. Table S5. Immune gene signatures. Table. S6. PI score and functional immune gene sets. Table S7. PI score and immune subsets estimated by hematoxylin and eosin staining. Table S8. PI score and immune subset estimate by gene signatures. Table S9. Survival and parainflammation. Table S10. Clinical features and parainflammation. Table S11. Primers used for qPCR analyses. (PDF 1608 kb) [file 13059_2016_995_MOESM3_ESM.pdf]

Supplementary Figure 1: qPCR of PI genes in organoids

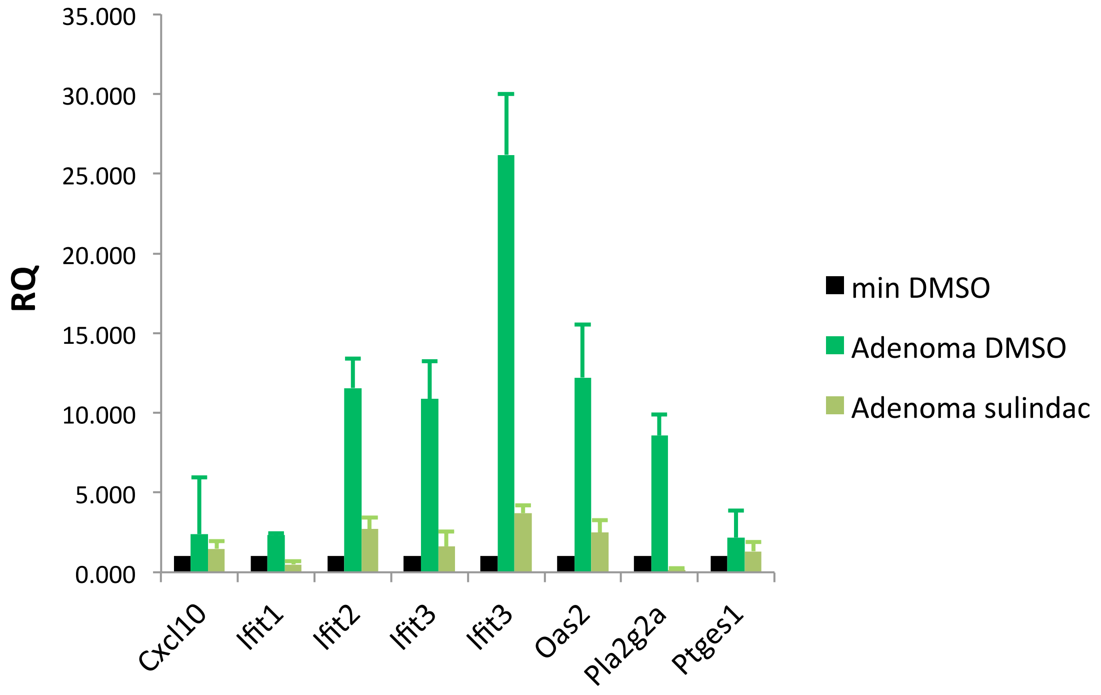

Figure S1. qPCR of PI genes in organoids. Organoids were generated from normal intestinal mucosa (min) or adenoma tissue (adenoma) of $APC^{min/+}$ mice. The organoids were treated with sulindac 500uM or DMSO for 48h. mRNA levels of PI genes were measured by qPCR.

Supplementary Figure 2: Expression of PI genes in cell lines.

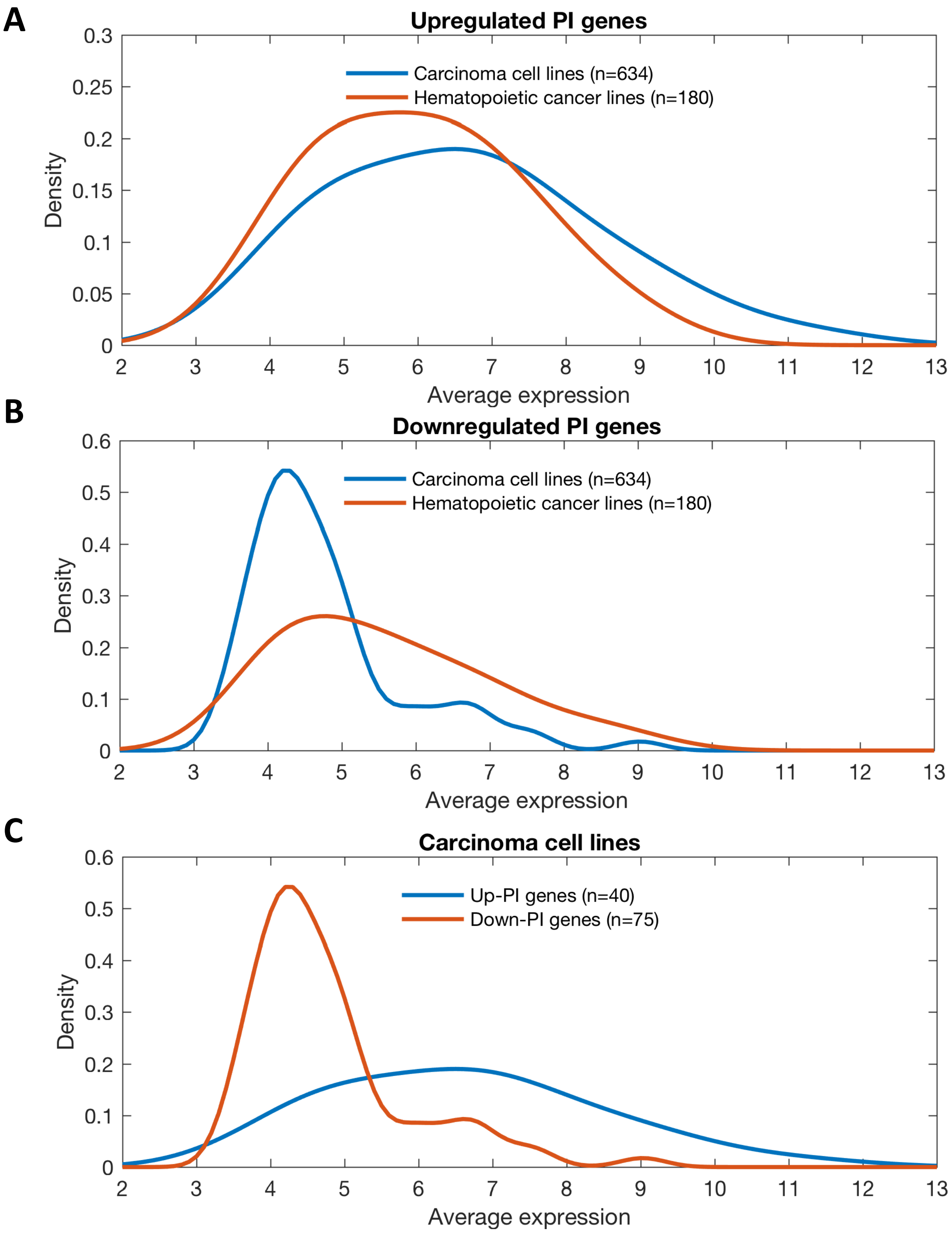

Figure S2. Expression of PI genes in cell lines. A) Distribution of the average expression levels (log2 RMA) of the 40 upregulated PI genes in carcinoma cell lines compared to hematopoietic cell lines using the CCLE data. PI genes are expressed in carcinomas more than in leukemias. **B)** Same, using the 75 downregulated PI genes. **C)** Comparison of the up and downregulated PI genes. The majority of the downregulated PI genes are not expressed in carcinoma cell lines.

Supplementary Figure 3: Correlation between the PI scores derived from up and downregulated genes.

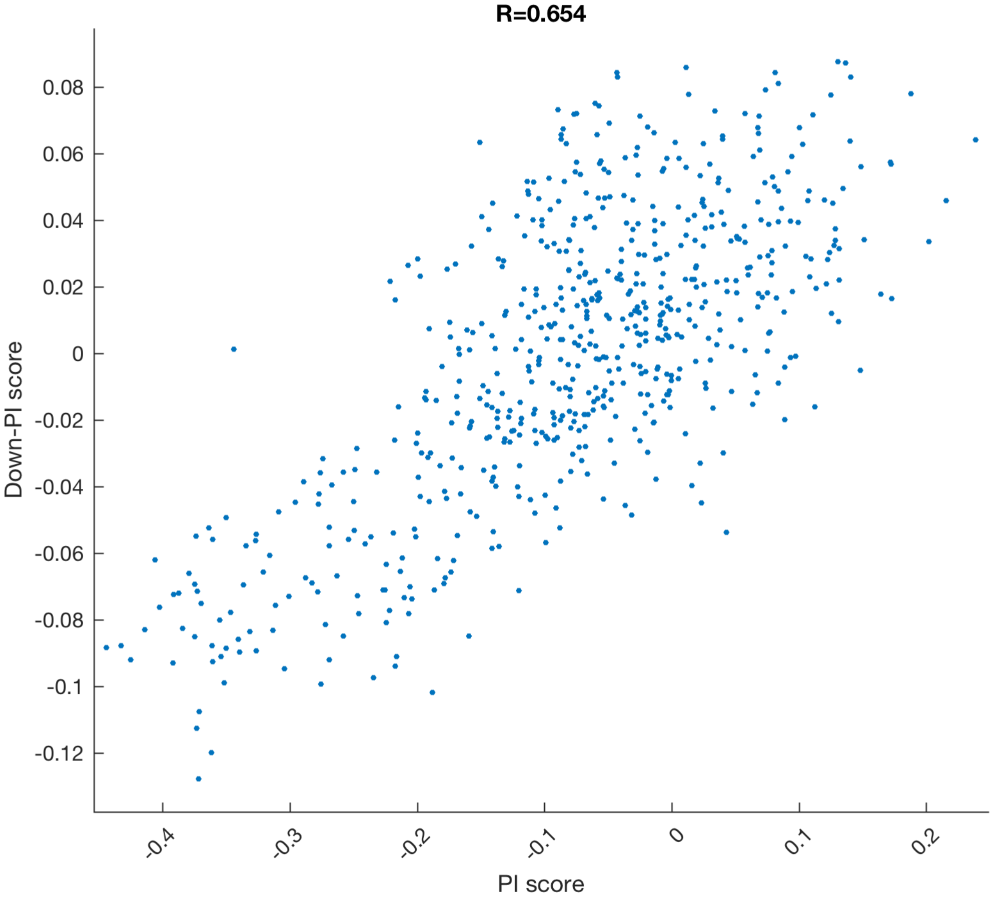

Figure S3. Correlation between the PI scores derived from up and downregulated genes. Scatter plot of ssGSEA PI scores (shifted such that PI+ is > 0) vs. ssGSEA scores derived from the 75 downregulated PI genes in 634 carcinoma cell lines. Spearman correlation is presented.

Supplementary Figure 4: Adjusting TCGA expression levels to immune infiltrations

A **CD45 is highly correlated with ESTIMATE immune score**

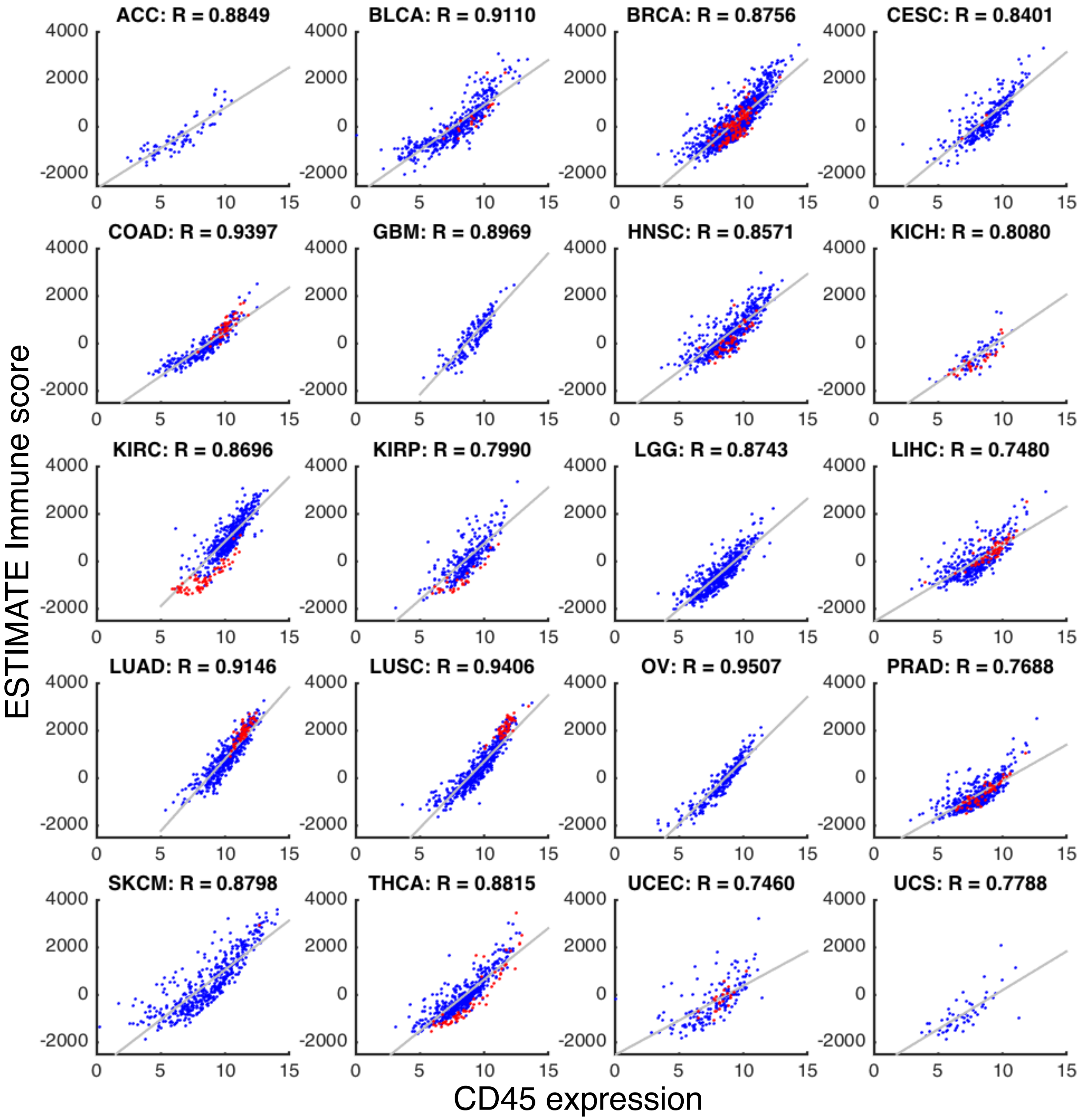

B Learning normal association of expression with immune estimates

TCGA cancer type	GTEx Tissue	# of GTEx samples
ACC	Adrenal Gland	52
BLCA	Bladder	11
BRCA	Breast	66
**CESC**	**Cervix Uteri**	**9**
COAD	Colon	74
GBM	Brain	357
HNSC	Esophagus	227
KICH	Kidney	8
KIRC	Kidney	8
KIRP	Kidney	8
LGG	Brain	357
LIHC	Heart	133
LUAD	Lung	133
LUSC	Lung	133
OV	Ovary	35
PAAD	Pancreas	65
PRAD	Prostate	42
**SKCM**	**Skin**	**322**
**THCA**	**Thyroid**	**120**
UCEC	Uterus	36
UCS	Uterus	36

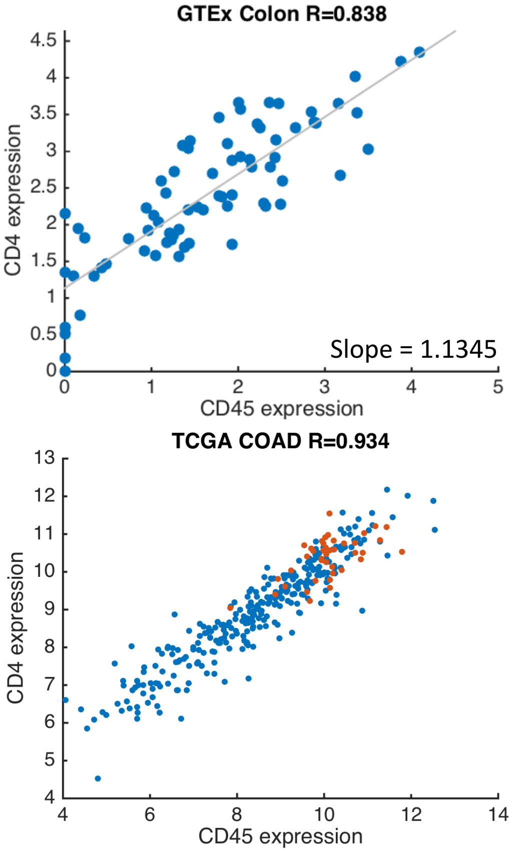

C Adjusting TCGA expression levels using the learned slope

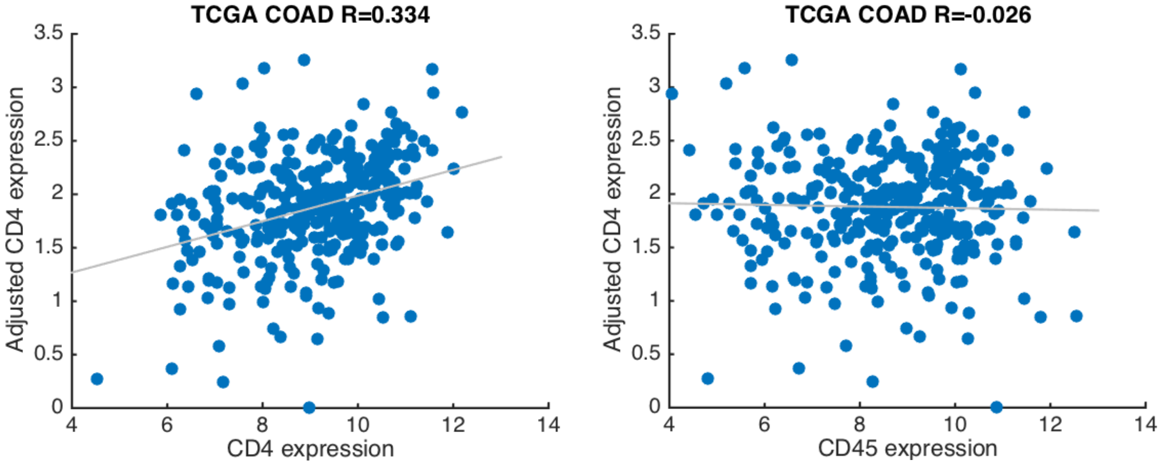

Figure S4. Adjusting TCGA expression levels to immune infiltrations. A. Scatter plots of CD45 (PTPRC) expression against the ESTIMATE immune score in each of the cancer types. Tumor samples are in blue, adjacent normal are in red. **B.** To adjust the TCGA samples we obtained expression data for normal samples from GTEx. The table shows the GTEx tissue and the number of samples that were used to learn the slopes in 21 cancer types. 3 cancer types were later omitted from further analyses due to high correlation with CD45 after adjustment (see figure S3). The left panel shows an example for the gene CD4 in colon. **C.** Using the slope learned in colon for CD4 we fit the expression levels of colon adenocarcinoma (COAD) TCGA samples. The adjusted expression is then shifted to 0. After adjustment there is no correlation with CD45 (right).

Supplementary Figure 5: Correlation of the PI score with CD45 expression

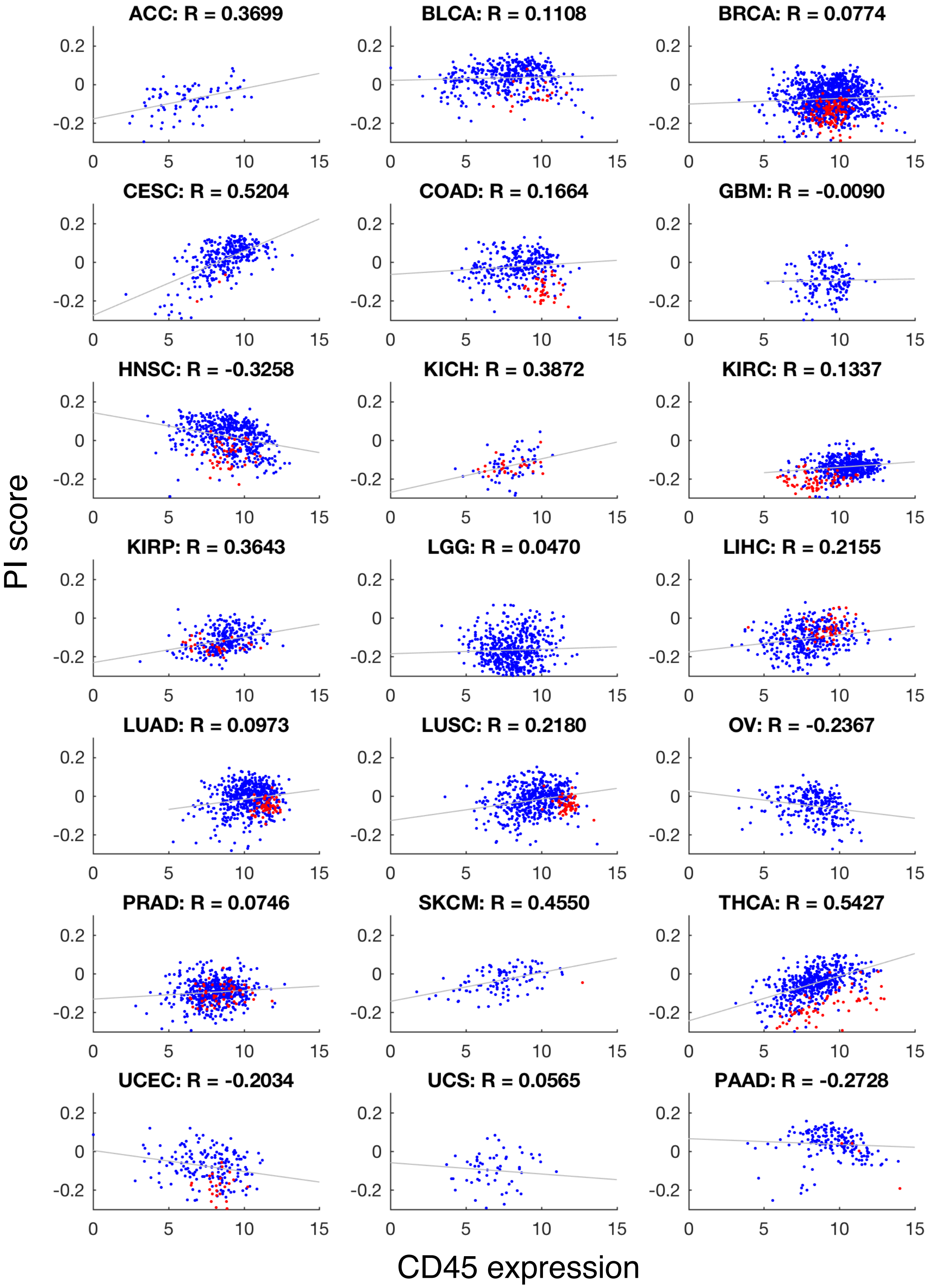

Figure S5. Correlation of the PI score with CD45 expression. PI scores (y-axis) vs. CD45 expression in 21 cancer types. Tumor samples are in blue, adjacent normal are in red. R is the spearman coefficient. In 3 cancer types the correlation of CD45 with the PI score remained high (|R|>0.4) even after adjustment: CESC, THCA and SKCM. This failure in adjustment is possibly due to bad fitting with GTEx tissue types. These cancer types were omitted from further analyses.

Supplementary Figure 6: PI scores in TCGA cancer and adjacent normal samples

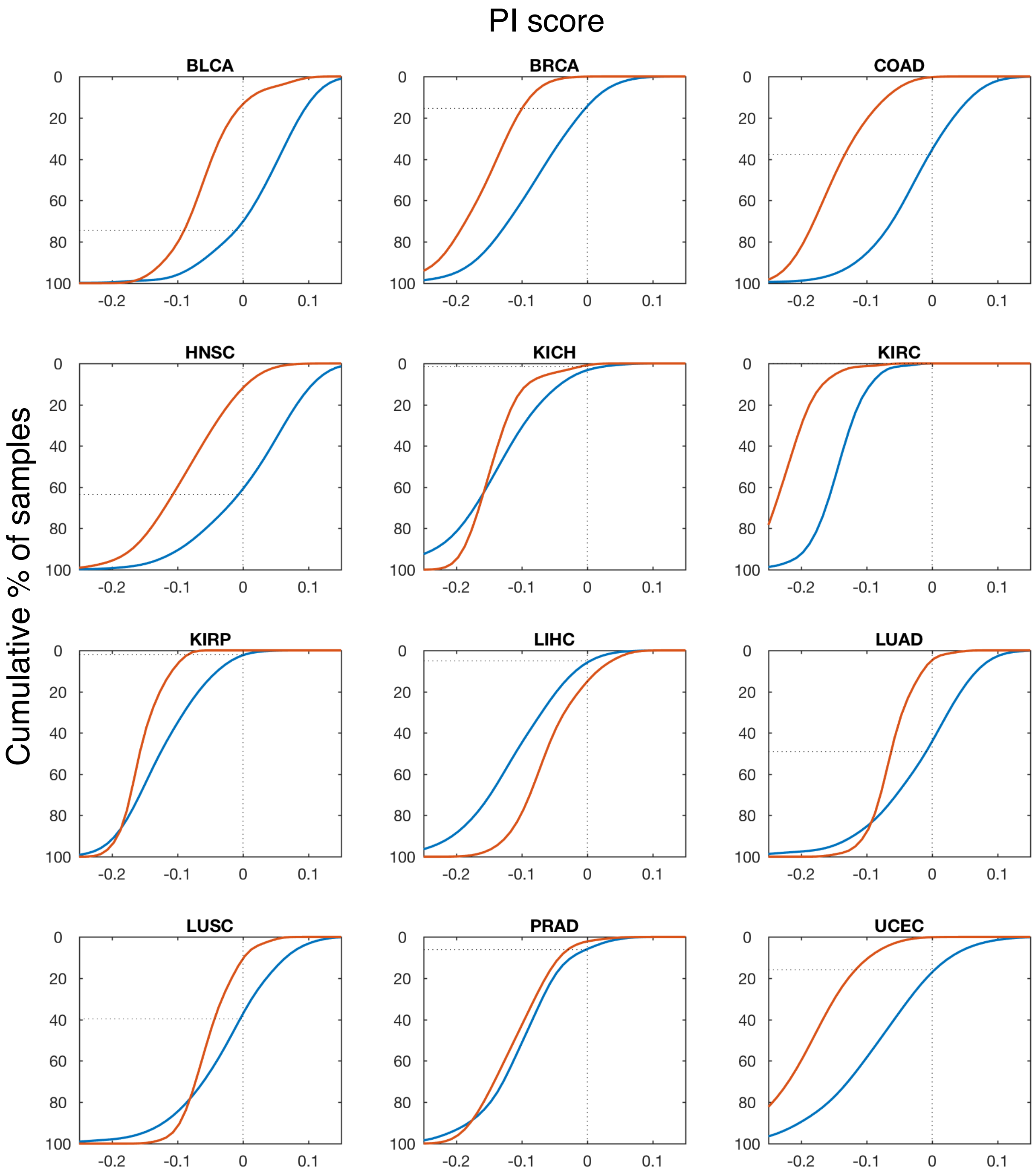

Figure S6. PI scores in TCGA cancer and adjacent normal samples. PI scores in tumors (blue) and adjacent normal (red) samples in 12 cancer types with at least 19 adjacent normal samples. The y-axis is the cumulative percent of samples over the score. Samples with positive score are PI+ (dashed line).

Supplementary Figure 7: The PI score is correlated with the number of activated PI genes.

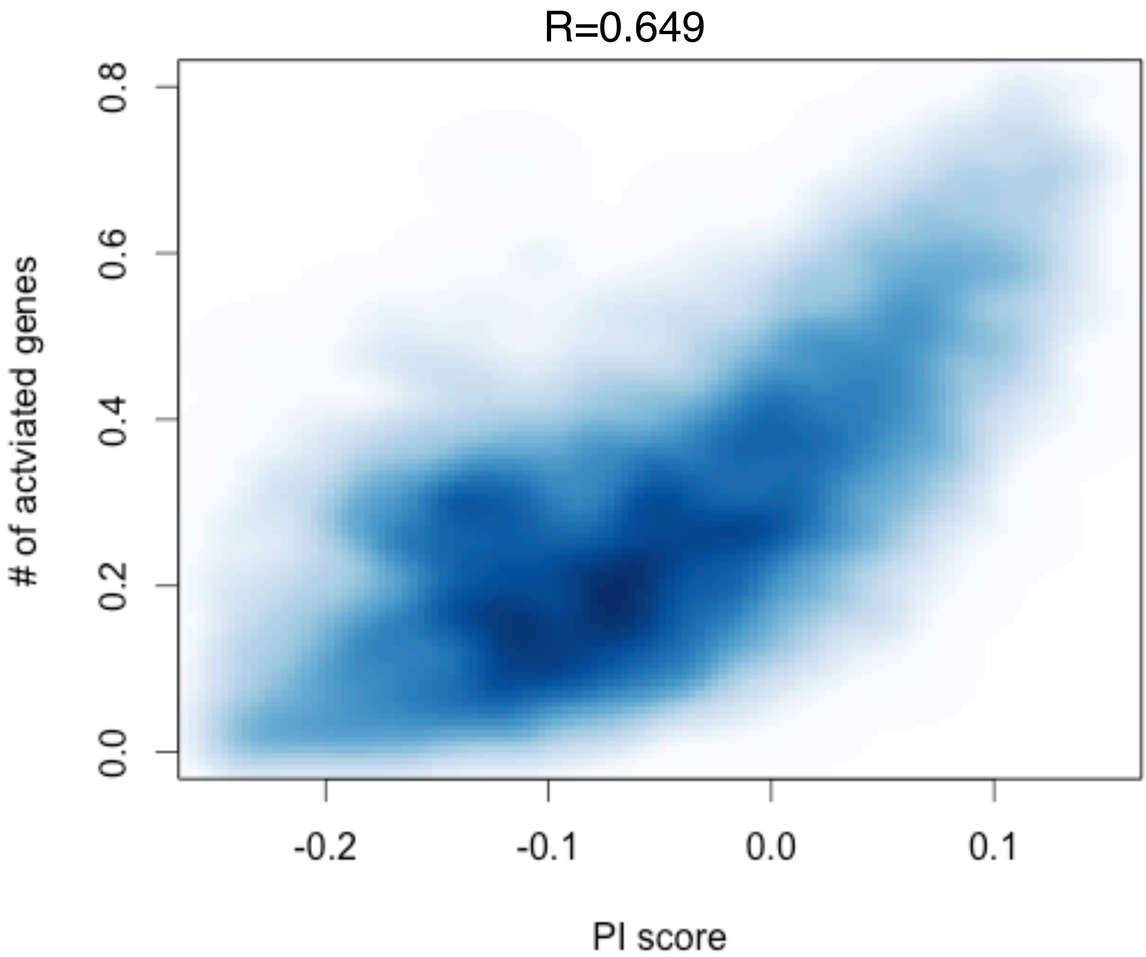

Figure S7. The PI score is correlated with the number of activated PI genes. The adjusted expression of the PI genes was normalized to 0 mean and 1 standard deviation. A gene is "activated" if the relative expression is > 0.5. Spearman coefficient is presented. Data points were smoothed using the 'smoothScatter' R package. The median number of 'activated' genes in PI+ samples is 17, compared to 8 in PI- samples. Moreover, only 3.7% of PI- samples have 17 or more 'activated' genes.

This analysis shows that the PI score, which is calculated using a single sample's gene expression profile, is also strongly associated with relative expression of the genes compared to all samples.

Supplementary Figure 8: PI-associated genes response to Aspirin treatment

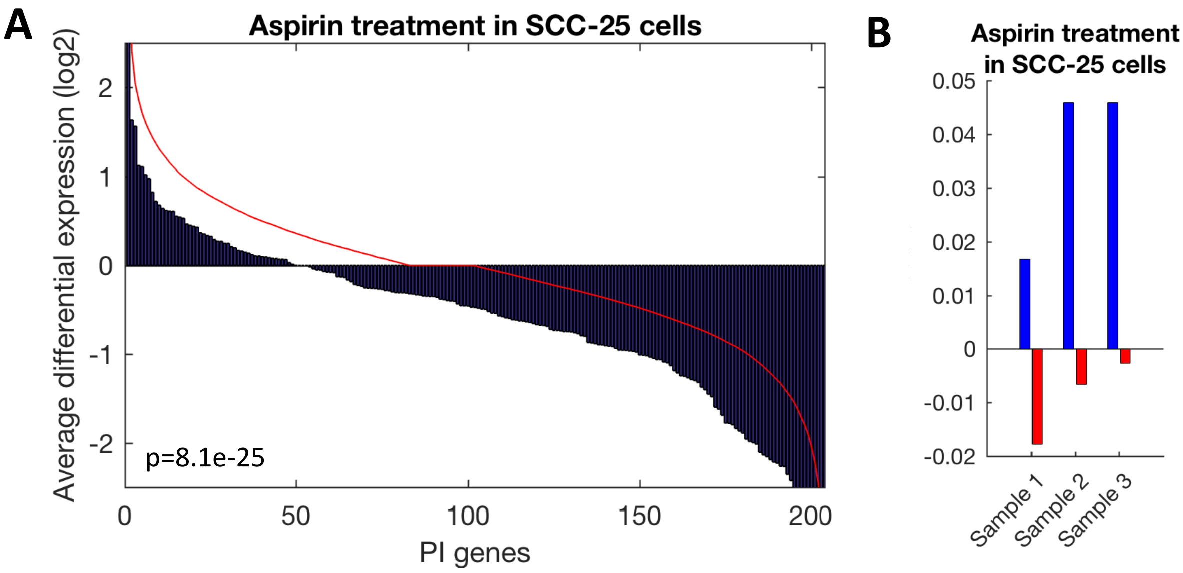

Figure S8. PI-associated genes response to Aspirin treatment. A. Average of the differential expression (in log2 scale) of the three replicates each of control and aspirin treated SCC-25 cells for the 215 PI-associated genes. A kernel smoothed regression curve for all genes is presented as reference (in red). All the PI-associated genes are downregulated after treatment compared to expected by random. The p-value presented was calculated using Wilcoxon rank-sum test. **B.** The PI score (using the 215 PI-associated genes) calculated for each sample before and after treatment.

Supplementary Table 1: Parainflammation gene signature

Mouse gene symbol	Human gene symbol	Entrez ID	Entrez Gene Name	Type(s)
Aim2	AIM2	9447	absent in melanoma 2	other
Anxa1	ANXA1	301	annexin A1	enzyme
Blnk	BLNK	29760	B-cell linker	other
Bst2	BST2	684	bone marrow stromal cell antigen 2	other
Ccnd1	CCND1	595	cyclin D1	transcription regulator
Cd14	CD14	929	CD14 molecule	transmembrane receptor
Cd276	CD276	80381	CD276 molecule	other
Cd44	CD44	960	CD44 molecule (Indian blood group)	enzyme
Cxcl10	CXCL10	3627	chemokine (C-X-C motif) ligand 10	cytokine
Cxcl9	CXCL9	4283	chemokine (C-X-C motif) ligand 9	cytokine
Hmox1	HMOX1	3162	heme oxygenase 1	enzyme
Icam1	ICAM1	3383	intercellular adhesion molecule 1	transmembrane receptor
Ifit1	IFIT1	3434	interferon-induced protein with tetratricopeptide repeats 1	other
Ifit2	IFIT2	3433	interferon-induced protein with tetratricopeptide repeats 2	other
Ifit3	IFIT3	3437	interferon-induced protein with tetratricopeptide repeats 3	other
Ifitm3	IFITM3	10410	interferon induced transmembrane protein 3	other
Il1rn	IL1RN	3557	interleukin 1 receptor antagonist	cytokine
Il33	IL33	90865	interleukin 33	cytokine
Isg15	ISG15	9636	ISG15 ubiquitin-like modifier	other
Itga2	ITGA2	3673	integrin, alpha 2 (CD49B, alpha 2 subunit of VLA-2 receptor)	transmembrane receptor
Lgmn	LGMN	5641	legumain	peptidase
Mmp7	MMP7	4316	matrix metallopeptidase 7	peptidase
Mx1	MX1	4599	MX dynamin-like GTPase 1	enzyme
Mx2	MX2	4600	MX dynamin-like GTPase 2	enzyme
Nox1	NOX1	27035	NADPH oxidase 1	ion channel
Oas1g	OAS1	4938	2'-5'-oligoadenylate synthetase 1, 40/46kDa	enzyme
Oas2	OAS2	4939	2'-5'-oligoadenylate synthetase 2, 69/71kDa	enzyme
Oas3	OAS3	4940	2'-5'-oligoadenylate synthetase 3, 100kDa	enzyme
Pla2g2a	PLA2G2A	5320	phospholipase A2, group IIA (platelets, synovial fluid)	enzyme
Pla2g2d	PLA2G2D	26279	phospholipase A2, group IID	enzyme
Plat	PLAT	5327	plasminogen activator, tissue	peptidase
Plaur	PLAUR	5329	plasminogen activator, urokinase receptor	transmembrane receptor
Pparg	PPARG	5468	peroxisome proliferator-activated receptor gamma	ligand-dependent nuclear receptor
Ptges	PTGES	9536	prostaglandin E synthase	enzyme
Rel	REL	5966	v-rel avian reticuloendotheliosis viral oncogene homolog	transcription regulator
Retnlb	RETNLB	84666	resistin like beta	other
Scarb1	SCARB1	949	scavenger receptor class B, member 1	transporter
Tirap	TIRAP	114609	toll-interleukin 1 receptor (TIR) domain containing adaptor protein	other
Tlr2	TLR2	7097	toll-like receptor 2	transmembrane receptor
Tnfrsf12a	TNFRSF12A	51330	tumor necrosis factor receptor superfamily, member 12A	transmembrane receptor

Table S1. Parainflammation gene signature. 40 inflammatory response genes that were upregulated in an RNA-seq analyses of two mouse models. The table presents also the human orthologs of the mouse PI genes.

Supplementary Table 2: Down-regulated parainflammation gene

Mouse gene symbol	Human gene symbol	Entrez ID	Entrez Gene Name	Type(s)
Abcg1	ABCG1	9619	ATP binding cassette subfamily G member 1	transporter
Ace	ACE	1636	angiotensin I converting enzyme	peptidase
Alox5ap	ALOX5AP	241	arachidonate 5-lipoxygenase-activating protein	other
Apoa1	APOA1	335	apolipoprotein A-I	transporter
Atf3	ATF3	467	activating transcription factor 3	transcription regulator
C1qa	C1QA	712	complement component 1, q subcomponent, A chain	other
C1qb	C1QB	713	complement component 1, q subcomponent, B chain	other
C1qc	C1QC	714	complement component 1, q subcomponent, C chain	other
C1s	C1S	716	complement component 1, s subcomponent	peptidase
Ccl24	CCL24	6369	chemokine (C-C motif) ligand 24	cytokine
Cd200	CD200	4345	CD200 molecule	other
Cd200r1	CD200R1	131450	CD200 receptor 1	other
Cd300lf	CD300LF	146722	CD300 molecule like family member f	other
Cd36	CD36	948	CD36 molecule	transmembrane receptor
Cd4	CD4	920	CD4 molecule	transmembrane receptor
Cd40lg	CD40LG	959	CD40 ligand	cytokine
Cd5l	CD5L	922	CD5 molecule like	transmembrane receptor
Cd69	CD69	969	CD69 molecule	transmembrane receptor
Cd8a	CD8A	925	CD8a molecule	other
Cish	CISH	1154	cytokine inducible SH2-containing protein	other
Cd209a	CLEC4M	10332	C-type lectin domain family 4 member M	other
Csf1r	CSF1R	1436	colony stimulating factor 1 receptor	kinase
Csf2	CSF2	1437	colony stimulating factor 2	cytokine
Cxcr6	CXCR6	10663	chemokine (C-X-C motif) receptor 6	G-protein coupled receptor
Cybb	CYBB	1536	cytochrome b-245, beta polypeptide	enzyme
Ets1	ETS1	2113	v-ets avian erythroblastosis virus E26 oncogene homolog 1	transcription regulator
Fasl	FASLG	356	Fas ligand	cytokine
Fcer1g	FCER1G	2207	Fc fragment of IgE receptor Ig	transmembrane receptor
Gbp1	GBP1	2633	guanylate binding protein 1, interferon-inducible	enzyme
Hck	HCK	3055	HCK proto-oncogene, Src family tyrosine kinase	kinase
Ido1	IDO1	3620	indoleamine 2,3-dioxygenase 1	enzyme
Il15	IL15	3600	interleukin 15	cytokine
Il16	IL16	3603	interleukin 16	cytokine
Il2rb	IL2RB	3560	interleukin 2 receptor subunit beta	transmembrane receptor
Il2rg	IL2RG	3561	interleukin 2 receptor subunit gamma	transmembrane receptor
Inpp5d	INPP5D	3635	inositol polyphosphate-5-phosphatase D	phosphatase
Irf4	IRF4	3662	interferon regulatory factor 4	transcription regulator
Itgam	ITGAM	3684	integrin subunit alpha M	transmembrane receptor
Itgb2	ITGB2	3689	integrin subunit beta 2	transmembrane receptor
Itgb7	ITGB7	3695	integrin subunit beta 7	transmembrane receptor
Jun	JUN	3725	jun proto-oncogene	transcription regulator
Lum	LUM	4060	lumican	other
Ly9	LY9	4063	lymphocyte antigen 9	other
Lyz2	LYZ	4069	lysozyme	enzyme
Mbl2	MBL2	4153	mannose-binding lectin (protein C) 2, soluble	other
Ncf1	NCF1	653361	neutrophil cytosolic factor 1	enzyme
Nckap1l	NCKAP1L	3071	NCK associated protein 1 like	other
Nlrp6	NLRP6	171389	NLR family, pyrin domain containing 6	G-protein coupled receptor
Nlrp9b	NLRP9	338321	NLR family, pyrin domain containing 9	other
Nr1h3	NR1xH3	10062	nuclear receptor subfamily 1 group H member 3	ligand-dependent nuclear receptor
Nt5e	NT5E	4907	5'-nucleotidase ecto	phosphatase
P2rx7	P2RX7	5027	purinergic receptor P2X, ligand gated ion channel, 7	ion channel
Pglyrp2	PGLYRP2	114770	peptidoglycan recognition protein 2	transmembrane receptor
Pik3cg	PIK3CG	5294	phosphatidylinositol-4,5-bisphosphate 3-kinase catalytic subunit gamma	kinase
Prf1	PRF1	5551	perforin 1 (pore forming protein)	other
Pros1	PROS1	5627	protein S (alpha)	other
Ptgs1	PTGS1	5742	prostaglandin-endoperoxide synthase 1 (prostaglandin G/H synthase and cyclooxygenase)	enzyme
Ptprc	PTPRC	5788	protein tyrosine phosphatase, receptor type C	phosphatase
Reg3b	REG3A	5068	regenerating family member 3 alpha	enzyme
Rgs1	RGS1	5996	regulator of G-protein signaling 1	other
Selplg	SELPLG	6404	selectin P ligand	other
Siglec5	SIGLEC8	27181	sialic acid binding Ig-like lectin 8	transmembrane receptor
Slamf7	SLAMF7	57823	SLAM family member 7	other
Spib	SPIB	6689	Spi-B transcription factor (Spi-1/PU.1 related)	transcription regulator
Stat4	STAT4	6775	signal transducer and activator of transcription 4	transcription regulator
Tbx21	TBX21	30009	T-box 21	transcription regulator
Tnfaip8l2	TNFAIP8L2	79626	TNF alpha induced protein 8 like 2	other
Traf1	TRAF1	7185	TNF receptor associated factor 1	other
Trat1	TRAT1	50852	T cell receptor associated transmembrane adaptor 1	kinase
Trim38	TRIM38	10475	tripartite motif containing 38	other
Tyrobp	TYROBP	7305	TYRO protein tyrosine kinase binding protein	transmembrane receptor
Vav1	VAV1	7409	vav guanine nucleotide exchange factor 1	transcription regulator
Vegfc	VEGFC	7424	vascular endothelial growth factor C	growth factor
Was	WAS	7454	Wiskott-Aldrich syndrome	other
Xcl1	XCL1	6375	chemokine (C motif) ligand 1	cytokine

Table S2. Down-regulated parainflammation genes. 75 inflammatory response genes that were downregulated in an RNA-seq analyses of two mouse models. The table presents also the human orthologs of the mouse genes.

Supplementary Table 3:
List of samples acquired from TCGA

TCGA abbreviation	Cancer type	Cancer #	Adjacent normal #
**ACC**	Adrenocortical carcinoma	79	0
**BLCA**	Bladder Urothelial Carcinoma	407	19
**BRCA**	Breast invasive carcinoma	1091	111
**COAD**	Colon adenocarcinoma	286	41
**GBM**	Glioblastoma multiforme	166	0
**HNSC**	Head and Neck squamous cell carcinoma	514	43
**KICH**	Kidney Chromophobe	66	25
**KIRC**	Kidney renal clear cell carcinoma	534	72
**KIRP**	Kidney renal papillary cell carcinoma	291	32
**LGG**	Brain Lower Grade Glioma	530	0
**LIHC**	Liver hepatocellular carcinoma	373	50
**LUAD**	Lung adenocarcinoma	513	58
**LUSC**	Lung squamous cell carcinoma	501	51
**OV**	Ovarian serous cystadenocarcinoma	265	0
**PAAD**	Pancreatic adenocarcinoma	178	4
**PRAD**	Prostate adenocarcinoma	497	52
**UCEC**	Uterine Corpus Endometrial Carcinoma	175	24
**UCS**	Uterine Carcinosarcoma	57	0
**CESC**	Cervical squamous cell carcinoma and endocervical adenocarcinoma	304	3
**SKCM**	Skin Cutaneous Melanoma	106	1
**THCA**	Thyroid carcinoma	501	59

Table S3. List of samples acquired from TCGA. Number of cancer and adjacent normal samples in 21 TCGA cancer types that were used in this study. Only primary tumor samples were analyzed. The 3 cancer types on the bottom were omitted from the analyses due to high correlation with CD45 after adjustment of expression to immune infiltrations (see supplementary figure 2). The data was downloaded from the TCGA portal on January 15, 2015.

Supplementary Table 4:
Transcription factors binding enrichment analysis of PI genes

Factor	Total Genes with Factor	Extended PI genes (207 genes)	Q-value (Hypergeometric Test; Benjamini-Hochberg)
Stat2Ifna6h	288	49	5.09E-45
Irf1Ifna30	171	40	7.05E-43
Stat1Ifna6h	319	45	8.56E-38
Stat1Ifna30	286	41	8.91E-35
Stat2Ifna30	742	46	1.14E-22
Stat3Tam112h	1620	64	3.31E-21
Stat3Etoh01c	1666	62	3.48E-19
Stat3Etoh01b	1649	61	9.14E-19
CfosTam14h	1265	52	8.36E-18
Prdm1	282	26	2.44E-17
Pol2	13720	190	2.85E-16
Stat3Tam	2217	67	2.85E-16
CfosTam112h	1621	54	1.22E-14
Stat3	1170	44	1.04E-13
CfosEtoh01	1478	47	5.86E-12
CfosTam	1769	49	2.74E-10
Cebpb	5485	98	1.44E-09
Taf1	11637	160	5.68E-09
Stat1Ifng30	2801	61	1.30E-08
Tbp	10552	149	1.30E-08
Batf	854	29	3.23E-08
Irf4	1671	43	3.81E-08
Stat3Etoh01	928	30	5.14E-08
Smc3	4062	76	5.96E-08
Irf1Ifna6h	3027	62	8.11E-08
Stat1Ifng6h	610	23	1.34E-07
P300	7444	112	8.21E-07
Pol2Etoh01	8593	121	7.73E-06
Baf155	1364	33	7.97E-06
Ap2alpha	3106	57	1.29E-05
Pol2Tam	8580	120	1.29E-05
Rad21	5777	88	3.34E-05
Ebf1	3943	66	4.02E-05
Ap2gamma	4170	67	1.33E-04
NfkbTnfa	6987	99	1.47E-04
Ikzf1	193	9	1.70E-04
Max	11417	144	1.70E-04
Yy1	10448	134	2.32E-04
Pu1	4392	68	3.37E-04
Cjun	2138	39	5.67E-04
Fosl2	3159	52	6.18E-04
CmycTam14h	2561	44	8.03E-04
Bcl11a	761	17	3.94E-03
Irf1Ifng30	4194	61	4.50E-03
eGFP-Fos	402	10	1.30E-02
Cfos	3726	53	1.59E-02
Znf217	208	6	2.41E-02
Gr	3749	52	2.81E-02
CmycEtoh01	5767	74	3.62E-02
Tcf7l2	6234	79	3.66E-02
Tcf12	5918	75	4.48E-02
Sin3a	10934	127	4.93E-02
Pol2b	5310	68	4.94E-02

Table S4. Transcription factors binding enrichment analysis of PI genes. The table presents the transcription factors (TFs) with significant enriched binding in promoters of genes associated with the PI score (R>0.5 in the CCLE dataset). The analysis was performed using the ENCODE ChIP-Seq Significance Tool (http://encodeqt.simple-encode.org/). Only 207 of the 215 PI-associated genes were matched with a symbol in the analysis.

Supplementary Table 5: Immune gene signatures

Gene set	Gene symbols	Source
**NF-kappaB Pathway**	ACP2,ACPP,CCNB1,DLGAP1,EGR3,ENPP2,FBXW11,GABRG2,GDF15,H1FX,HMGCR,ITPKA,IVD,KIAA0196,RAB8A,RNF2,SPINT1,TPX2	Jin*
**TNF Signaling**	ADAM17,BAG4,CASP8,CYLD,FADD,OTUD7B,RIPK1,TNF,TNFRSF1A,TRADD,TRAF2,USP2,USP21,USP4	Reactome
**Type I Interferon Response**	MX1,TNFSF10,RSAD2,IFIT1,IFIT3,IFIT2,IRF7,DDX4,MX2,ISG20	Rooney***
**Type II Interferon Response**	GPR146,SELP,AHR	Rooney***
**B cells**	CD79B,BTLA,FCRL3,BANK1,CD79A,BLK,RALGPS2,FCRL1,HVCN1,BACH2	Rooney***
**CD4+ Regulatory T cells**	FOXP3,C15orf53,IL5,CTLA4,IL32,GPR15,IL4	Rooney***
**CD8+ T cells**	CD8A	Rooney***
**Macrophages**	FUCA1,MMP9,LGMN,HS3ST2,TM4SF19,CLEC5A,GPNMB,C11orf45,CD68,CYBB	Rooney***
**Neutrophils**	KDM6B,HSD17B11,EVI2B,MNDA,MEGF9,SELL,NLRP12,PADI4,TRANK1,VNN3	Rooney***
**NK cells**	KLRF1,KLRC1	Rooney***
**pDCs**	LILRA4,CLEC4C,PLD4,PHEX,IL3RA,PTCRA,IRF8,IRF7,GZMB,CXCR3	Rooney***

* Jin, Renjie, et al. "NF-κB gene signature predicts prostate cancer progression." Cancer research 74.10 (2014): 2763-2772.
** Milacic, Marija, et al. "Annotating cancer variants and anti-cancer therapeutics in reactome." Cancers 4.4 (2012): 1180-1211. APA
*** Rooney, Michael S., et al. "Molecular and genetic properties of tumors associated with local immune cytolytic activity." Cell 160.1 (2015): 48-61. APA

Table S5. Immune gene signatures. Lists of genes used to calculate enrichment scores of immune functional sets and immune subsets.

Supplementary Table 6: PI score and functional immune gene sets.

	Type I IFN Reponse	TNF-signaling	NF-kappaB signaling	Type II IFN Reponse	Cytolytic Activity
**PAAD**	0.8812	0.5332	0.2713	-0.0135	-0.1618
**BLCA**	0.77	0.3245	-0.0244	-0.2167	0.4057
**HNSC**	0.8452	0.4944	0.1303	-0.2304	0.3692
**LUAD**	0.7419	0.1594	0.2848	0.0764	0.0982
**LUSC**	0.8055	0.27	0.1607	0.0067	0.2995
**COAD**	0.7914	0.173	0.0272	0.0942	0.4293
**OV**	0.8183	0.3886	0.3239	0.163	0.0762
**UCEC**	0.8144	0.3438	0.2414	0.067	0.2766
**BRCA**	0.8367	0.239	0.136	-0.1352	0.232
**GBM**	0.7534	0.3968	0.137	0.0384	0.37
**ACC**	0.7081	0.1606	0.0551	-0.0282	0.3728
**UCS**	0.7943	0.394	0.2114	0.2704	0.4714
**PRAD**	0.7525	0.188	-0.1197	0.095	0.3243
**LIHC**	0.6735	0.0268	0.0803	0.2423	0.1744
**LGG**	0.7207	0.3617	0.1915	0.1928	0.5642
**KIRP**	0.3019	-0.278	0.463	-0.0383	0.5388
**KICH**	0.6238	-0.0379	0.1031	-0.2019	0.5221
**KIRC**	0.4827	0.138	0.3329	-0.0074	0.2194
**CCLE**	**0.8532**	**0.5267**	**0.1285**	**0.4185**	**-0.0943**

Table S6. PI score and functional immune gene sets. Spearman coefficients for the correlations of cancer type's functional immune gene sets scores calculated using ssGSEA and the PI score. The gene signatures can be found in supplementary table 5. High correlations are observed with type I interferon (IFN) signaling.

Supplementary Table 7: PI score and immune subsets estimated by Hematoxylin and Eosin

	Lymphocytes	Monocytes	Neutrophils
**BLCA**	0.0448	0.0575	0.0586
**BRCA**	0.0553	0.0522	0.0693
**COAD**	0.1852	-0.1055	0.1612
**HNSC**	-0.1116	0.0419	0.0646
**KIRC**	0.0728	0.0126	0.0424
**KIRP**	0.1518	0.3255	0.0489
**LGG**	0.1176	0.0362	0
**LIHC**	0.1075	0.019	0.044
**LUAD**	-0.0359	-0.0396	-0.0823
**LUSC**	0.1575	-0.0123	0.1071
**PRAD**	0.0527	0.0934	0.085
**UCEC**	-0.0296	0.0133	0.0749
**PAAD**	-0.069	0.0162	-0.0476

Table S7. PI score and immune subsets estimated by Hematoxylin and Eosin. Spearman coefficients for the correlations of cancer types with at least 80 samples of estimation of major immune subsets based on Hematoxylin and Eosin (H&E) statining and PI scores. All correlations are lower than 0.2.

Supplementary Table 8: PI score and immune subsets estimate by gene signatures

	Macrophages		pDCs		CD4+ Regulatory T-cells		Neutrophils		NK cells		B-cells		CD8+ T-cells	
	**TCGA**	**CCLE**	**TCGA**	**CCLE**	**TCGA**	**CCLE**	**TCGA**	**CCLE**	**TCGA**	**CCLE**	**TCGA**	**CCLE**	**TCGA**	**CCLE**
**BLCA**	0.4436	0.5354	0.3196	0.3126	0.3673	0.0617	0.0897	0.1007	0.2883	-0.1508	-0.0145	0.0824	0.2298	0.0177
**BRCA**	0.3596	0.4915	0.2729	-0.0844	0.2709	0.3745	0.1434	0.3831	0.0944	-0.2573	-0.0091	-0.2912	0.1204	-0.2379
**COAD**	0.42	0.353	0.3233	0.2733	0.2657	0.2198	0.2888	0.3845	0.2724	-0.0049	-0.0023	0.1418	0.3726	-0.0682
**HNSC**	0.1254	0.4364	0.1094	0.5635	0.0192	0.5044	0.0159	0.0605	0.0345	-0.1589	-0.3316	0.182	0.1461	-0.2071
**KIRC**	0.3114	0.2666	0.2932	-0.1426	0.2207	0.2675	0.0945	-0.0203	0.1266	0.1966	0.1214	0.123	0.2055	0.0746
**LIHC**	0.4842	0.6123	0.4289	-0.0085	0.4755	0.1877	0.2534	0.4215	0.202	0.0421	0.3006	0.2191	0.3229	-0.1631
**LUAD**	0.4206	0.433	0.3241	0.3701	0.1154	0.6387	0.1994	0.4497	0.118	0.1865	-0.0481	0.3899	0.1062	-0.1776
**LUSC**	0.4258	0.1237	0.2646	0.0515	0.3362	0.1401	0.1869	0.0416	0.1447	0.0684	0.0279	-0.1314	0.2109	-0.3924
**OV**	0.1792	0.3157	0.1536	0.4584	0.0124	0.2203	0.0978	0.2608	0.0626	0.0188	-0.2056	-0.1211	-0.1039	-0.4392
**PAAD**	0.4491	0.422	0.0057	-0.0815	0.0443	0.4042	-0.1088	0.3541	-0.2099	-0.1665	-0.2331	0.4132	-0.2405	-0.0822
**UCEC**	0.3624	0.4829	0.2683	0.3578	0.1522	0.7179	0.175	-0.3724	-0.0358	-0.2228	-0.0581	0.0031	0.0442	-0.1233

Table S8. PI score and immune subsets estimate by gene signatures. Spearman coefficients for the correlations of cancer type's immune subsets scores calculated using ssGSEA and the PI score for both TCGA and CCLE samples. The gene signatures can be found in supplementary table 5. Only cancer types with at least 20 samples in CCLE are presented. We observed same trends in both TCGA and CCLE across cancer types, suggesting that the correlations are not a result of immune infiltrations, rather activation of shared pathways of PI with macrophages, pDCs and regulatory T-cells.

Supplementary Table 9: Survival and parainflammation

Cancer type	N	Cox p-value	Beta	% PI+	% died	Log-rank p-value	Median survival PI+ (days)	Median survival PI- (days)	Age controlled p-value	Age + smoking controlled p-value
**PAAD**	177	6.7E-05	8.3195	77.97	33.33	3.2E-04	591	2110	2.3E-04	5.4E-04
**KIRC**	533	5.9E-04	7.3887	0	30.02	N/A	N/A	N/A	1.5E-03	8.7E-02
**LGG**	515	8.4E-04	4.6645	3.69	17.86	8.5E-04	756	2649	2.8E-02	N/A
**HNSC**	511	1.4E-03	3.5242	63.6	32.29	4.1E-03	1131	1748	1.8E-03	8.3E-04
**LUAD**	492	9.4E-03	3.469	49.19	24.59	9.8E-03	1147	1503	7.6E-03	8.0E-03
**UCEC**	174	5.9E-02	7.2108	16.09	7.47	6.8E-01	N/A	2959	3.7E-02	N/A
**GBM**	159	2.5E-01	1.3922	11.32	66.67	9.1E-01	418	397	5.0E-01	N/A
**PRAD**	496	2.7E-01	-5.6807	6.05	1.61	9.3E-01	N/A	N/A	2.7E-01	N/A
**LUSC**	491	3.2E-01	1.1492	39.71	31.57	6.3E-01	1324	1636	2.6E-01	2.8E-01
**UCS**	57	3.7E-01	1.8476	8.77	56.14	7.3E-01	N/A	708	4.3E-01	N/A
**BLCA**	406	3.8E-01	-1.1843	74.63	26.11	7.9E-01	1842	1042	6.9E-01	7.0E-01
**KIRP**	287	4.1E-01	2.7741	2.09	11.15	8.2E-01	N/A	N/A	4.5E-01	1.6E-01
**ACC**	79	6.1E-01	1.5126	8.86	31.65	2.4E-03	552	N/A	6.6E-01	N/A
**BRCA**	1084	6.2E-01	-0.7571	15.5	9.32	3.5E-01	3446	3916	7.7E-01	N/A
**LIHC**	370	6.6E-01	0.763	4.86	24.05	9.9E-01	1746	1694	5.6E-01	N/A
**OV**	259	7.0E-01	-0.4589	22.39	56.37	2.3E-01	1419	1252	9.6E-01	N/A
**KICH**	65	7.8E-01	-1.3429	1.54	12.31	8.0E-01	1140	N/A	8.1E-01	5.4E-01
**COAD**	282	9.3E-01	0.1933	38.3	14.54	9.5E-01	2856	2361	9.4E-01	1.0E+00

Table S9. Survival and parainflammation. Survival analysis performed for each cancer type. The table presents the number of samples with both PI score and survival data, Cox regression p-value of the survival predicted by PI score and the beta of the regression. A positive beta means that there is higher mortality is associated with higher PI score. In 11 of the cancer types beta>1 and only in 3 beta<-1. Additionally, we performed a log-rank analysis of PI+ vs. PI-, and median survival is presented as well. Finally, we performed Cox regression analysis controlling the PI score to age at diagnosis and age + a smoking indicator (current smoker, stopped smoking <15 years ago, stopped smoking >15 years ago, non-smoker). The significance of PI score in predicting survival in PAAD, LGG, HNSC and LUAD is maintained in all analyses.

Supplementary Table 10: Clinical features and parainflammation

`	Age	Gender	Race	Weight	Height	Pathologic T	Pathologic N	Pathologic M	Pathologic stage	Smoking history
**ACC**	9.5E-02	7.0E-01	N/A	N/A	N/A	4.9E-01	6.1E-01	N/A	2.2E-01	N/A
**BLCA**	7.9E-01	8.4E-01	9.4E-01	9.8E-01	3.9E-01	5.6E-01	5.4E-01	4.6E-01	2.9E-01	5.8E-01
**BRCA**	8.3E-01	2.4E-01	5.6E-03	N/A	N/A	6.2E-01	8.1E-02	6.6E-01	8.2E-01	N/A
**COAD**	8.9E-02	3.8E-01	3.6E-02	4.1E-01	9.0E-01	8.5E-01	3.8E-01	2.5E-01	4.3E-01	N/A
**GBM**	5.4E-01	5.8E-01	7.3E-01	N/A	N/A	N/A	N/A	N/A	N/A	N/A
**HNSC**	7.7E-01	3.0E-03	6.4E-02	N/A	N/A	3.8E-04	2.2E-01	6.8E-01	9.5E-01	1.2E-01
**KICH**	1.8E-01	1.4E-01	N/A	N/A	N/A	3.5E-01	4.5E-01	6.2E-01	1.2E-01	N/A
**KIRC**	1.4E-01	9.4E-01	5.1E-03	N/A	N/A	2.5E-03	1.1E-01	1.3E-03	1.1E-05	9.6E-01
**KIRP**	9.3E-02	9.6E-01	5.0E-02	4.3E-02	4.4E-01	1.1E-01	1.1E-01	7.3E-01	2.6E-01	8.7E-01
**LGG**	4.4E-07	3.2E-01	6.2E-01	N/A	N/A	N/A	N/A	N/A	N/A	N/A
**LIHC**	4.7E-01	6.6E-06	3.6E-01	7.4E-02	4.3E-03	4.8E-02	1.2E-01	9.2E-02	1.8E-01	N/A
**LUAD**	6.2E-01	2.8E-03	2.3E-02	N/A	N/A	6.3E-02	5.3E-03	1.7E-01	3.1E-02	1.0E-01
**LUSC**	3.4E-01	4.9E-01	2.3E-01	N/A	N/A	5.1E-01	7.9E-01	4.2E-01	6.0E-01	1.2E-01
**OV**	1.6E-03	N/A	3.9E-01	N/A	N/A	N/A	N/A	N/A	N/A	N/A
**PAAD**	5.2E-02	6.0E-01	1.2E-01	N/A	N/A	9.9E-04	1.6E-02	3.1E-01	3.2E-05	3.3E-01
**PRAD**	5.6E-01	N/A	7.1E-02	N/A	N/A	5.3E-01	3.1E-03	N/A	N/A	N/A
UCEC	8.5E-01	N/A	5.7E-01	2.7E-01	8.5E-01	N/A	N/A	N/A	N/A	N/A
UCS	6.7E-1	N/A	7.1E-1	5.9E-01	8.8E-01	N/A	N/A	N/A	N/A	N/A

Table S10. Clinical features and parainflammation. P-values of the associations between the PI scores and different clinical features. P-value of binary or categorical clinical feature were calculated using one-way analysis of variance (ANOVA); continuous clinical features were calculated with Spearman correlation. Significant associations (p-value < 0.01) are marked in red).

Supplementary Table 11: Primers used for qPCR analyses.

Human		
Gene	Forward primer	Reverse primer
AIM2	GGCTTTGGTTTTGTTACCGA	GCAGTGATGAAGACCATTCG
BLNK	AGAGATGTACGTGATGCCCG	CCTCTGGCTTGATCGATTGTCTAT
BST2	GGAAGCTGGCACATCTTGGA	CTAACCGTGTTGCCCCATGA
CXCL9	AGTGCAAGGAACCCCAGTAG	AGGGCTTGGGGCAAATTGTT
CXCL10	AGCAGAGGAACCTCCAGTCT	AGGTACTCCTTGAATGCCACT
IFI44	TGGGAGCTGGACCCTGTAAA	CCTCCCTTAGATTCCCTATTTGCT
IFITM3	CCGTGAAGTCTAGGGACAGG	CCTGGAAGATCAGCACTGGG
MMP7	GTCTCTGGACGGCAGCTATG	GATAGTCCTGAGCCTGTTCCC
MX1	ACCATTCCAAGGAGGTGCAG	TGCGATGTCCACTTCGGAAA
MX2	TGACCTCATCGACTCCCTGC	GTTACGATTCCGCTGCCTCT
OAS2	GCTCAGAAGCTGGGTTGGTT	GAGCCACCTATGGCCACTC
OAS3	CCAGCAGTGTACCAAGATCTCC	CTGCTCCCAGGCATACACAG
UBC	ATTTGGGTCGCGGTTCTTG	TGCCTTGACATTCTCGATGGT
Mouse		
Gene	Forward primer	Reverse primer
CXCL10	GAAGCTTGAAATCATCCCTGC	ACGCTTTCATTAAATTCTTGATGG
IFIT1	CCCAGAGAACAGCTACCACCTT	TGCTCTGAGATTCTCACTTCCAA
IFIT2	AGCAGACAGTTACACAGCAGTCAT	TCCTCAAACTCATCCAAGGACT
IFIT3	TTCTGAACTGCTCAGCCCA	TGACTGGACATACTTCCTTCCCT
IFITM3	CTGCTGCCTGGGCTTCATAG	GGATGCTGAGGACCAAGGTG
OAS2	TGGAAAGTGCCAGTAATGCAG	TCCAGAAGCGTCTTCCAGAG
PLA2G2A	AAGGCCTTGAACAAGAAACCA	GCCATGATCGAGGCTGCTA
UBC	CAGCCGTATATCTTCCCAGACT	CTCAGAGGGATGCCAGTAATCTA
